# Misrepresentation of Randomized Controlled Trials in Press Releases and News Coverage: A Cohort Study

**DOI:** 10.1371/journal.pmed.1001308

**Published:** 2012-09-11

**Authors:** Amélie Yavchitz, Isabelle Boutron, Aida Bafeta, Ibrahim Marroun, Pierre Charles, Jean Mantz, Philippe Ravaud

**Affiliations:** 1 INSERM, U738, Paris, France; 2 Centre d'Épidémiologie Clinique, AP-HP (Assistance Publique des Hôpitaux de Paris), Hôpital Hôtel Dieu, Paris, France; 3 Université Paris Descartes, Sorbonne Paris Cité, Faculté de Médecine, Paris, France; 4 Department of Internal Medicine, Hôpital Foch, Suresnes, France; 5 Department of Anesthesiology and Critical Care, Beaujon University Hospital, Clichy, France; University of California, San Francisco, United States of America

## Abstract

**Background:**

Previous studies indicate that in published reports, trial results can be distorted by the use of “spin” (specific reporting strategies, intentional or unintentional, emphasizing the beneficial effect of the experimental treatment). We aimed to (1) evaluate the presence of “spin” in press releases and associated media coverage; and (2) evaluate whether findings of randomized controlled trials (RCTs) based on press releases and media coverage are misinterpreted.

**Methods and Findings:**

We systematically searched for all press releases indexed in the EurekAlert! database between December 2009 and March 2010. Of the 498 press releases retrieved and screened, we included press releases for all two-arm, parallel-group RCTs (*n* = 70). We obtained a copy of the scientific article to which the press release related and we systematically searched for related news items using Lexis Nexis.

“Spin,” defined as specific reporting strategies (intentional or unintentional) emphasizing the beneficial effect of the experimental treatment, was identified in 28 (40%) scientific article abstract conclusions and in 33 (47%) press releases. From bivariate and multivariable analysis assessing the journal type, funding source, sample size, type of treatment (drug or other), results of the primary outcomes (all nonstatistically significant versus other), author of the press release, and the presence of “spin” in the abstract conclusion, the only factor associated, with “spin” in the press release was “spin” in the article abstract conclusions (relative risk [RR] 5.6, [95% CI 2.8–11.1], *p*<0.001). Findings of RCTs based on press releases were overestimated for 19 (27%) reports. News items were identified for 41 RCTs; 21 (51%) were reported with “spin,” mainly the same type of “spin” as those identified in the press release and article abstract conclusion. Findings of RCTs based on the news item was overestimated for ten (24%) reports.

**Conclusion:**

“Spin” was identified in about half of press releases and media coverage. In multivariable analysis, the main factor associated with “spin” in press releases was the presence of “spin” in the article abstract conclusion.

## Abstract 2

A study conducted by Amélie Yavchitz and colleagues examines the factors associated with “spin” (specific reporting strategies, intentional or unintentional, that emphasize the beneficial effect of treatments) in press releases of clinical trials.

## Editors' Summary

**Background:**

The mass media play an important role in disseminating the results of medical research. Every day, news items in newspapers and magazines and on the television, radio, and internet provide the general public with information about the latest clinical studies. Such news items are written by journalists and are often based on information in “press releases.” These short communications, which are posted on online databases such as EurekAlert! and sent directly to journalists, are prepared by researchers or more often by the drug companies, funding bodies, or institutions supporting the clinical research and are designed to attract favorable media attention to newly published research results. Press releases provide journalists with the information they need to develop and publish a news story, including a link to the peer-reviewed journal (a scholarly periodical containing articles that have been judged by independent experts) in which the research results appear.

**Why Was This Study Done?:**

In an ideal world, journal articles, press releases, and news stories would all accurately reflect the results of health research. Unfortunately, the findings of randomized controlled trials (RCTs—studies that compare the outcomes of patients randomly assigned to receive alternative interventions), which are the best way to evaluate new treatments, are sometimes distorted in peer-reviewed journals by the use of “spin”—reporting that emphasizes the beneficial effects of the experimental (new) treatment. For example, a journal article may interpret nonstatistically significant differences as showing the equivalence of two treatments although such results actually indicate a lack of evidence for the superiority of either treatment. “Spin” can distort the transposition of research into clinical practice and, when reproduced in the mass media, it can give patients unrealistic expectations about new treatments. It is important, therefore, to know where “spin” occurs and to understand the effects of that “spin”. In this study, the researchers evaluate the presence of “spin” in press releases and associated media coverage and analyze whether the interpretation of RCT results based on press releases and associated news items could lead to the misinterpretation of RCT results.

**What Did the Researchers Do and Find?:**

The researchers identified 70 press releases indexed in EurekAlert! over a 4-month period that described two-arm, parallel-group RCTs. They used Lexis Nexis, a database of news reports from around the world, to identify associated news items for 41 of these press releases and then analyzed the press releases, news items, and abstracts of the scientific articles related to each press release for “spin”. Finally, they interpreted the results of the RCTs using each source of information independently. Nearly half the press releases and article abstract conclusions contained “spin” and, importantly, “spin” in the press releases was associated with “spin” in the article abstracts. The researchers overestimated the benefits of the experimental treatment from the press release as compared to the full-text peer-reviewed article for 27% of reports. Factors that were associated with this overestimation of treatment benefits included publication in a specialized journal and having “spin” in the press release. Of the news items related to press releases, half contained “spin”, usually of the same type as identified in the press release and article abstract. Finally, the researchers overestimated the benefit of the experimental treatment from the news item as compared to the full-text peer-reviewed article in 24% of cases.

**What Do These Findings Mean?:**

These findings show that “spin” in press releases and news reports is related to the presence of “spin” in the abstract of peer-reviewed reports of RCTs and suggest that the interpretation of RCT results based solely on press releases or media coverage could distort the interpretation of research findings in a way that favors experimental treatments. This interpretation shift is probably related to the presence of “spin” in peer-reviewed article abstracts, press releases, and news items and may be partly responsible for a mismatch between the perceived and real beneficial effects of new treatments among the general public. Overall, these findings highlight the important role that journal reviewers and editors play in disseminating research findings. These individuals, the researchers conclude, have a responsibility to ensure that the conclusions reported in the abstracts of peer-reviewed articles are appropriate and do not over-interpret the results of clinical research.

**Additional Information:**

Please access these Web sites via the online version of this summary at http://dx.doi.org/10.1371/journal.pmed.1001308.

- The PLOS Hub for Clinical Trials, which collects PLOS journals relating to clinical trials, includes some other articles on “spin” in clinical trial reports
- EurekAlert is an online free database for science press releases
- The UK National Health Service Choices website includes Beyond the Headlines, a resource that provides an unbiased and evidence-based analysis of health stories that make the news for both the public and health professionals
- The US-based organization HealthNewsReview, a project supported by the Foundation for Informed Medical Decision Making, also provides expert reviews of news stories

## Introduction

The media play an important role in the dissemination of findings from health research. More than half of US adults report that they follow health news closely [1]. Further, 90% of the general public gets most of its information about science from the mass media [2]. Press releases are a major source of information for one-third of medical reports in US newspapers [3]. Press releases are widely used by the medical researchers to attract favorable media attention [4]–[6] and to promote their research [7]–[9]. A press release should provide journalists with the basic information needed to develop a news story and publish it in the mass media.

Randomized controlled trials (RCTs) are considered the gold standard for therapeutic evaluation [10]. Adequate and undistorted communication of the findings from RCTs is essential for physicians, researchers, and patients because it allows for efficient uptake of research into clinical practice [11]. Theoretically, in reports of RCTs published in peer-reviewed journals, the data should speak for themselves. However, a recent study showed that research findings can be distorted in published articles, by the use of “spin,” which is defined as specific reporting emphasizing the beneficial effect of the experimental treatment [12]. The types of distorted presentation or “spin” are diverse, with, for example, a particular focus on statistically significant results (within-group comparison, subgroup analyses, and secondary outcomes) or an inadequate interpretation of nonstatistically significant differences as demonstrating equivalence in treatment effectiveness or lack of difference in adverse events.

We aimed to (1) evaluate the presence of “spin” in press releases and associated media coverage and (2) evaluate whether findings of RCTs contained within press releases and media coverage are misinterpreted.

## Methods

### Selection of Press Releases, Related Scientific Articles, and News Items

We identified all press releases indexed in EurekAlert! (online free database for science press releases; www.eurekalert.org) between December 1, 2009, and March 31, 2010, using the following search strategy: topic “medicine and health,” type of release “research news,” keyword: random* [4],[13]. We included press releases for published results of two-arm, parallel-group RCTs defined as prospective studies assessing health care interventions in human participants. To have a homogeneous sample, we excluded press releases for equivalence or noninferiority, cross-over, cluster, and multiple-arm trials; follow-up studies; press releases not reported in English; and those about more than one study. Duplicate press releases (i.e., press releases published more than once in the database) were systematically searched and excluded.

The title and full text of all retrieved press releases were screened by one reviewer to exclude any non-eligible press releases.

We obtained a copy of the scientific article related to the press release from (1) the direct link or full reference citation reported in the press release, if available; or (2) the PubMed single citation matcher indicating the year of publication, journal, and author's name. Each retrieved scientific article (abstract and full text) was assessed by the same reader to confirm eligibility.

Finally, for all selected press releases, we systematically searched for related news items in the “general news” library of LEXIS-NEXIS using (1) the name of the disease; (2) the treatment being evaluated, and, if needed, the name of the first or second author. All news related to the articles or press releases were retrieved, and we selected the news that had the highest number of words dedicated to the selected study.

### Data Abstraction

Data were abstracted from the press release, news items, and the related published scientific article. For this purpose, we developed a standardized data-abstraction form using previous work on the same topics [12]–[14]. The data-abstraction form and details about the methods is available in Texts S1 and S2.

The data-abstraction form was preliminarily tested by two of the reviewers with a sample of 15 press releases and original articles indexed in January 2008. The data that involved some subjectivity, such as the type of “spin” were abstracted by two independent reviewers, with discrepancies resolved by consensus. Other data were evaluated by a single reviewer. The concordance between the two reviewers for the assessment of “spin” is reported in Text S3; the mean kappa coefficient for “spin” was 0.56 (range 0.43–0.69).

We systematically extracted data related to the characteristics of (1) the RCT, (2) the press releases, and (3) the presence of “spin” in the article abstract conclusions, in the press release and, when available, in the news items.

We defined “spin” as a specific reporting (intentional or unintentional) that emphasizes the beneficial effect of the experimental treatment. We used a classification of “spin” described in a previous work [12]. This classification was initially developed in the context of trials with a nonstatistically significant primary outcome. This classification was adapted for all RCTs. We considered “spin” as being a focus on statistically significant results (within-group comparison, secondary outcomes, subgroup analyses, modified population of analyses); an interpretation of statistically nonsignificant results for the primary outcomes as showing treatment equivalence or comparable effectiveness; or any inadequate claim of safety or emphasis of the beneficial effect of the treatment.

### Results Interpretation

The RCT results were interpreted independently from three different sources: (1) from the full text of the scientific article, (2) from the press release, and (3) from the news items.

For each source, different pairs of assessors independently evaluated the results of the RCT and achieved consensus. Assessment based on the scientific article relied on the results for the primary outcomes, secondary outcomes, and harm. For assessment of press releases, assessors were blinded to the authors of the press release, the content of the scientific article, and the journal of publication. For assessment of news items, assessors were blinded to the content of the press release and scientific article. All results reported represent the consensus of each pair of assessors.

#### Interpreting the RCT results

The trial results were interpreted independently by use of the same scale, from 1 to 5 [15]. According to this scale, the assessors had to indicate whether patients should (1) definitely get the experimental treatment evaluated, (2) probably get the experimental treatment evaluated, (3) decide for themselves (i.e., the article was neutral), (4) probably not get the experimental treatment evaluated, or (5) definitely not get the experimental treatment evaluated. If the interpretation of the RCT results was classified as 1 or 2, the experimental treatment was considered beneficial; 3, the trial results were neutral; 4 or 5, the experimental treatment was considered not beneficial.

#### Definition of misinterpretation

Misinterpretation was defined as the interpretation of the press release or news items differing from that based on the full-text article by at least one class according to the above three-class system of scores. Misinterpretation of the press release or news items could overestimate the treatment beneficial effect or underestimate the treatment effect. For example, an overestimation of the treatment beneficial effect in the press release or news items occurred when reading the published article led to rating the trial results as neutral, whereas reading the press release or new items led to rating the experimental treatment as beneficial.

### Statistical Analysis

Data for quantitative variables are expressed with medians and IQRs. Data for qualitative variables are expressed with frequencies and percentages. We planned bivariate and multivariable analysis to identify factors associated with (1) “spin” in the press releases, (2) an overestimation of the beneficial effect of the experimental treatment from press releases, (3) “spin” in the news items, and (4) an overestimation of the beneficial effect of the experimental treatment from news items. For bivariate analysis, we used the chi-square or Fisher exact test for categorical data and the Student *t*-test for quantitative data. For the multivariable analysis, we performed a Poisson regression with robust error variance [16] with a bootstrap model selection variable method [17] to assess all relevant variables. We used 1,000 bootstrap samples. Variables with *p*<0.25 in bivariate analysis were selected for possible inclusion in the multivariable model. Variables identified as independent factors associated with “spin” in the press release in at least 60% of the bootstrap samples were kept in the multivariable model.

Results are expressed as risk ratio (RR) and 95% CIs. We did not perform multivariable analysis to identify factors associated with overestimation of the benefit of the experimental treatment because there were few events as compared with the number of variables to include.

Statistical analysis involved use of SAS v9.1 (SAS Institute).

## Results

### Selection of Press Releases and Scientific Articles

**Figure 1:**
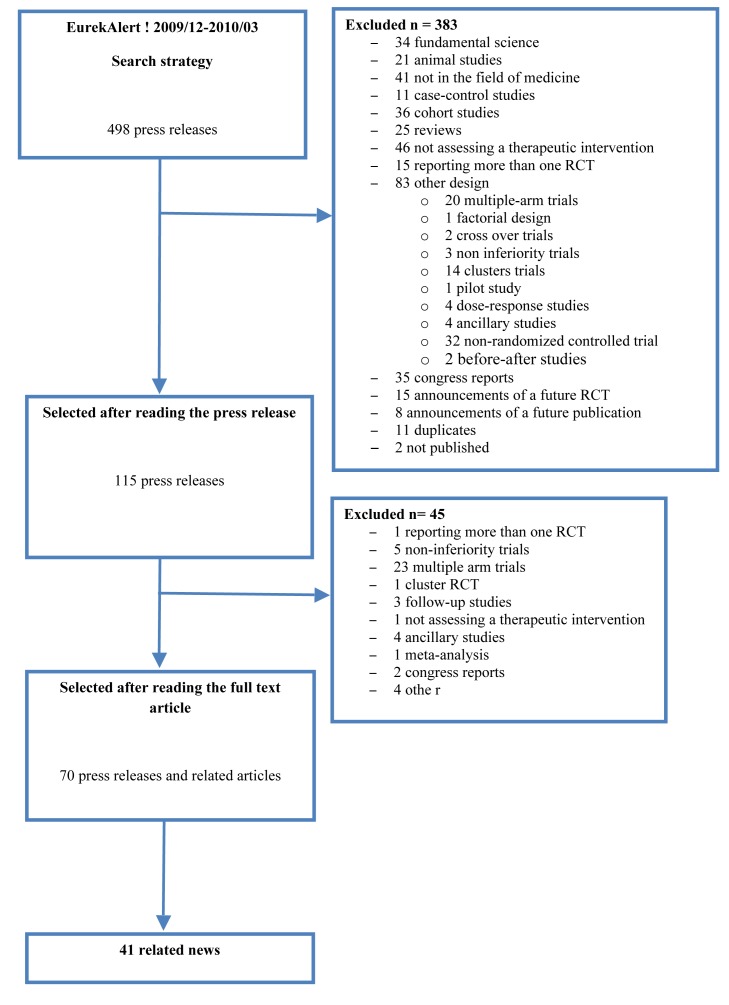
Flow diagram of the selected press releases and related articles.

The search strategy in EurekAlert! between December 1, 2009, and March 31, 2010, retrieved 498 press releases. The selection process resulted in 70 press releases and related scientific articles (Figure 1). Of these, 41 had associated news items. The list of press releases and published articles included is available in Text S4.

### Characteristics of RCTS

The description of the scientific articles is in Table 1. In total, 38 (54%) articles were published in a specialized journal; the median (interquartile range) journal impact factor was 17.2 (4.8–28.4). The funding source was for-profit (only or with a nonprofit source) for about half of the reports. In 34 reports (49%), the primary outcomes were statistically significant, and in 24 (34%), all primary outcomes were not statistically significant. In all, 28 articles (40%) exhibited at least one type of “spin” in the abstract conclusions. The main types of “spin” in the abstract conclusions were no acknowledgement of nonstatistically significant primary outcomes (20%); interpreting *p*>0.05 as demonstrating equivalence (7%); inappropriate extrapolation (9%); focus on statistically significant results such as subgroup analyses (6%), within-group comparisons (9%), and secondary outcomes (4%); or inadequate claim of safety (6%).

**Table 1:** General characteristics of articles.

Characteristics	Subcharacteristics	*n* = 70 (%)
Type of journal, *n* (%)	General medical journal	32 (46)
	Specialized medical journal	38 (54)
Funding source, *n* (%)	Profit or both profit and nonprofit	33 (47)
	None or nonprofit	33 (47)
	Not reported	4 (6)
Sample size median; [IQR]; (min–max)		112; [54–435]; (16–94,370)
Experimental treatment, *n* (%)	Drug	36 (51)
	Surgery/procedure	9 (13)
	Device	5 (7)
	Therapeutic strategy	7 (10)
	Participative intervention	12 (17)
	Other	1 (1)
Comparator, *n* (%)	Placebo	29 (41)
	Active treatment	32(46)
	Other	9 (13)
Primary outcomes clearly identified, *n* (%)		61(87)
Type of primary outcomes, *n* (%)	Efficacy	61 (87)
	Safety	1 (1)
	Both	4 (6)
	Unclear	4 (6)
Primary outcomes reported adequately, *n* (%)^a^		56 (80)
Results of primary outcomes, *n* (%)	All statistically significant	34 (49)
	All statistically nonsignificant	24 (34)
	Some statistically significant/some not	11 (16)
	Unclear	1 (1)
At least one “spin”		28 (40)
Type of “spin”^b^	No acknowledgment of nonstatistically significant primary outcome	14 (20)
	Claiming equivalence when results failed to demonstrate a statistically significant difference	5 (7)
	Focus on positive secondary outcome	3 (4)
	Focus on inappropriate subgroup	4 (6)
	Focus on within-group (or over-all within) comparison	6 (9)
	Nonstatistically significant outcome reported as if they were significant	3 (4)
	Ignored data of safety	1 (1)
	Inadequate claim of safety	4 (6)
	Inappropriate extrapolation	6 (9)
	Other	5 (7)

a Adequately, with effect size and precision or treatment effect in each arm with precision.

b Numbers do not add up as the types of “spin” were not mutually exclusive.

### Characteristics of Press Releases

The general characteristics of press releases are in Table 2: 57% were written by a press officer; half provided easy access to the research article that had been press released (i.e., a direct link or the full reference) and 25 (36%) reported the funding source. The results for primary outcomes were reported with words only in 29 (41%) press releases. Safety was mentioned in 24 (34%) and quantified in 14 (20%); the study limitations were reported in ten (14%). A total of 58 (83%) press releases contained quotations from authors or editors of the article. In 30 (52%), the interview reported results with emphasis, such as “this work paves the way for further study,” or in 22 (38%) with moderation, such as “further investigation is needed to establish (…).” Quotations from the article were included in 22 (31%) of the releases. In 11 (50%), the quotations reported results with emphasis, such as “clinical findings are indeed very encouraging, said Dr…”), or in seven (32%) with moderation.

**Table 2:** General characteristics of press releases.

Characteristics	Subcharacteristics	*n* = 70 (%)
Origin, *n* (%)	Press officer	40 (57)
	Industry or institution	30 (43)
Easy access to full article, (i.e., direct link or the full reference) *n* (%)		36 (51)
Funding reported, *n* (%)		25 (36)
Design reported, *n* (%)		70 (100)
Sample size reported, *n* (%)		65 (93)
Length of follow-up reported, *n* (%)		46 (66)
Primary outcomes reported, *n* (%)	In words only	29 (41)
	Per arms	30 (43)
	With effect size	17 (24)
Safety reported, *n* (%)	Mentioned	24 (34)
	Quantified	14 (20)
Limits reported, *n* (%)		10 (14)
Interview included, *n* (%)	Authors only	40 (57)
	Experts or editorialists only	6 (9)
	Both	12 (17)
Article quotation reported, *n* (%)		22 (31)
At least one type of “spin”		33 (47)
Type of “spin”^a^	No acknowledgment of nonstatistically significant primary outcome	13 (19)
	Claiming equivalence when results failed to demonstrate a statistically significant difference	7 (10)
	Focus on positive secondary outcome	5 (7)
	Focus on inappropriate subgroup	4 (5)
	Focus on within-group (or over-all within) comparison	11 (16)
	Nonstatistically significant outcome reported as if they were significant	5 (7)
	Ignored data of safety	3 (4)
	Inadequate claim of safety	5 (7)
	Inappropriate extrapolation	6 (9)
	Other “spin”	2 (3)

a Numbers do not add up as the types of “spin” were not mutually exclusive.

About half of the press releases (33; 47%) had at least one type of “spin” (Table 2).

### Factors Associated with “Spin” in Press Releases

From bivariate analysis (Table 3), “spin” in press releases was more frequent in trials published in a specialized journal (58% versus 34% in a general journal; *p* = 0.05), trials with small sample size (i.e., <112) (63% versus 31%; *p* = 0.008), and trials with “spin” in the scientific article abstract conclusion (93%, yes, versus 17%, no; *p*<0.001). The presence of “spin” in the press release was not associated with funding source (45% profit versus 49% other: *p* = 0.8), author of the press release (48% press officer versus 47% other; *p* = 0.9), the experimental treatment (47% drug versus 47% other; *p* = 1.0) or results of the primary outcome (46% all nonstatistically significant versus 48% other; *p* = 0.9). In multivariable analysis including all variables with *p*<0.25 in the bivariate analysis (i.e., journal, “spin” in the abstract conclusion, and sample size), the only factor associated with “spin” in the press release was “spin” in the scientific article abstract conclusions (RR = 5.6, 95% CI 2.8–11.0, *p*<0.001) (Text S5).

**Table 3:** Bivariate analysis of factors associated with and “spin” in the press releases.

Characteristics	Subcharacteristics	“Spin” in Press Release *n*/Total *n* (%)	*p*-Value
Journal	General	11/32 (34)	0.05
	Specialized	22/38 (58)	—
Funding source	Profit	15/33 (45)	0.8
	Nonprofit or not reported	18/37 (49)	—
Sample size	<112	22/35 (63)	0.008
	≥112	11/35 (31)	—
Experimental treatment	Drug	17/36 (47)	1.0
	Other	16/34 (47)	—
Results of primary outcome(s)	All nonstatistically significant	11/24 (46)	0.9
	Other	22/46 (48)	—
Authors of press release	Press officer	19/40 (48)	0.9
	Other	14/30 (47)	—
“Spin” in abstract conclusion	Yes	26/28 (93)	<0.001
	No	7/42 (17)	—

### Interpretation of the Trial Results from Press Releases

For the interpretation based on the full-text scientific articles, for 38 articles (54%), the experimental treatment was considered beneficial, 18 (26%) neutral, and 14 (20%) not beneficial. In contrast, for the interpretation based on press releases, for 55 releases (79%), the experimental treatment was considered beneficial, two (3%) neutral, and 13 (18%) not beneficial. The results were misinterpreted in 22 press releases (31%); for 19 (86%), the assessors overestimated the benefit of the experimental treatment from the press release and for three (14%), they underestimated the benefit of the experimental treatment from the press release.

As shown in Table 4, on the basis of press releases, the benefit of the experimental treatment was overestimated more often for trial results published in a specialized journal rather than in a general medical journal (45% versus 6%; *p*<0.001), for trials with a small rather than large sample size (46% versus 9%; *p*<0.001), for trials with nonstatistically rather than significant primary outcomes (42% versus20%; *p* = 0.05), and for trials with “spin” rather than without “spin” in the press release (48% versus 8%; *p*<0.001). These results did not differ significantly by funding source, author of the press release, or type of experimental treatment.

**Table 4:** Bivariate analysis of factors associated with an overestimation of the benefit of the experimental treatment from the press releases as compared with the interpretation from articles.

Characteristics	Subcharacteristics	Overestimation of the Benefit of the Experimental Treatment n/Total *n* (%)	*p*-Value
Journal	General	2/32 (6)	<0.001
	Specialized	17/38 (45)	
Funding source	Profit	7/33 (21)	0.3
	Nonprofit or not reported	12/37 (32)	
Sample size	*n*<112	16/35 (46)	<0.001
	*n*≥112	3/35 (9)	
Experimental treatment	Drug	11/36 (31)	0.5
	Other	8/34 (24)	
Results of primary outcome(s)	All nonstatistically significant	10/24 (42)	0.05
	Other	9/46 (20)	
Authors of press release	Press officer	10/40 (25)	0.6
	Other	9/30 (30)	
“Spin” in press releases	Yes	16/33 (48)	<0.001
	No	3/37 (8)	

### “Spin” and Interpretation of the News

For a sample of 41 RCTS we retrieved the scientific article, the press release, and any news items. “Spin” was identified in 17 (41%) abstracts, 19 (46%) press releases, and 21 (51%) news items.

**Figure 2:**
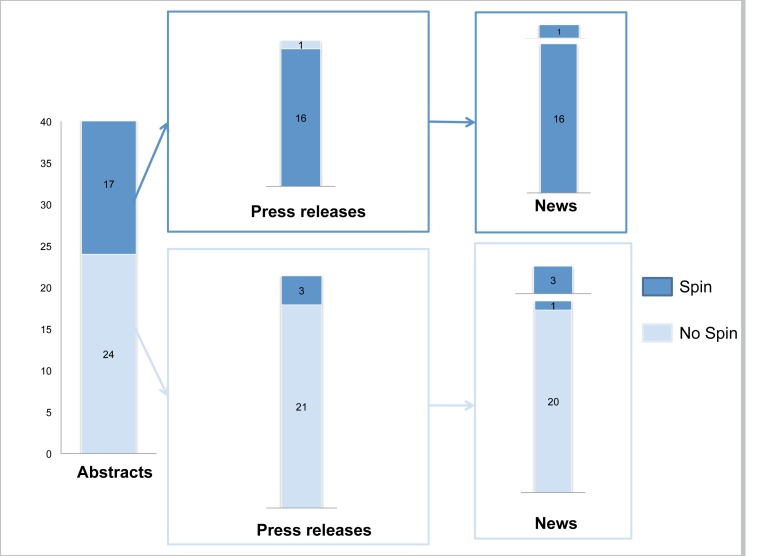
“Spin” in abstract conclusions, press releases, and news items.

**Figure 3:**
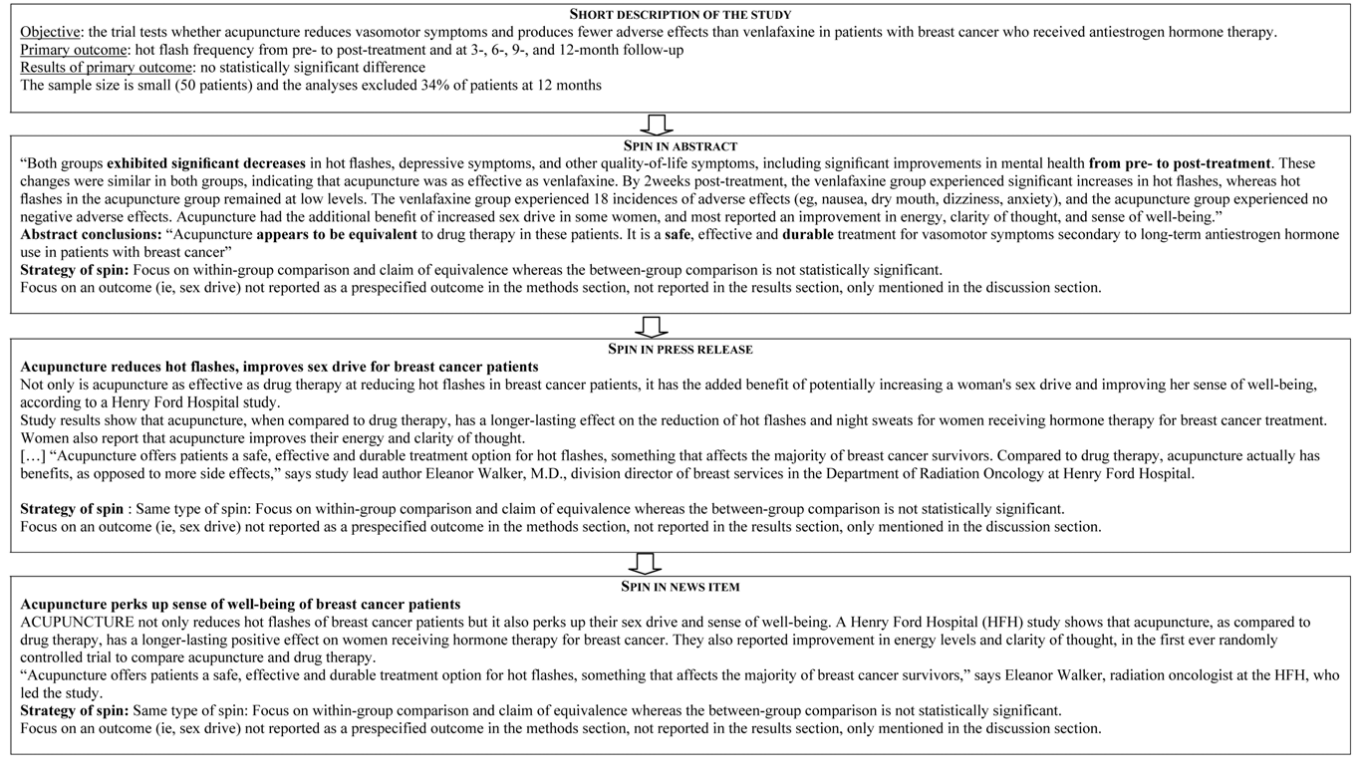
Examples of “spin” in abstracts, in press releases, and in related news items.

Figure 2 describes the reporting of “spin” in abstracts, press releases, and news items. For the 17 abstracts reported with “spin”, 16 press releases and related news items featured the same “spin.” For the 24 abstracts without “spin,” only three press releases featured “spin,” which was subsequently reported in the related news items. Examples of “spin” in the abstract and related press releases and news items are in Figure 3. The factors associated with “spin” in the news were specialty journals (67% versus 35%; *p* = 0.04), small sample size (68% versus 32%; *p* = 0.02), “spin” in abstract (100% versus 5%; *p*<0.001), and “spin” in the press release (100% versus 13%; *p*<0.001) (Text S6).

Overall, the assessors overestimated the benefit of the experimental treatment from the news for 10 (24%) reports. Factors associated with overestimation of the beneficial effect of the treatment from the news items were small sample size (41% versus 5%, *p* = 0.01), and “spin” in the news (43% versus 5%, *p* = 0.009) (Text S7).

## Discussion

Our results highlight a tendency for press releases and the associated media coverage of RCTs to place emphasis on the beneficial effects of experimental treatments. This tendency is probably related to the presence of “spin” in conclusions of the scientific article's abstract. This tendency, in conjunction with other well-known biases such as publication bias, selective reporting of outcomes, and lack of external validity, may be responsible for an important gap between the public perception of the beneficial effect and the real effect of the treatment studied.

Previous studies have highlighted the importance of press releases for results communication and dissemination [4],[5]. Indeed, as a direct means of communication between medical journals and the media, press releases provide an opportunity for journals to influence how the research is translated into news [4]. The press release is essential when considering the impact of press coverage by the media on health care utilization, clinical practice, and researchers' behavior [1]. This influence has been clearly shown in a quasi-experimental study evaluating the impact of media coverage [11]. The authors compared the number of scientific citations of articles published in the *New England Journal of Medicine* that were covered by the *New York Times* to similar articles that were not covered. They also performed this comparison during a 3-mo period when the *New York Times* was on strike; the *New York Times* continued to print an “edition of record” but did not sell copies to the public because of the strike. The authors demonstrated that the high citation of articles covered by the *New York Times* was not present during the strike. Consequently, the high citation was related to the media coverage, not the importance of the research [18]. A Cochrane systematic review highlighted the impact of the mass media on health services utilization [19]. It showed a consistent effect after planned campaigns and unplanned coverage. Another study showed a clear association of the media coverage of invasive group A streptococcal (GAS) disease and testing for GAS in pediatric emergency departments, with an important increase in the prescription of rapid tests for GAS in pediatric emergency departments concomitant with a peak in media attention, despite no increase in the number of children presenting symptoms that might warrant such testing [20].

Unfortunately, as shown in our study, and previous work the quality of media reports is questionable. An assessment of the reporting of medical news in the mainstream media highlighted the inadequate accuracy and balance of the news media in reporting medical science [21]–[23]. The criticisms of the mainstream media also applied to press releases. Woloshin et al., in evaluating press releases issued by 20 academic medical centers, showed that the releases frequently promoted preliminary research without giving basic details or the cautions needed to judge the meaning, relevance, or validation of the science (42% of press releases evaluated in this study did not provide any relevant caveats, and 90% about animal or laboratory studies lacked caveats about extrapolating results to humans) [13]. Furthermore, press releases tended to overstate the importance of the research, 29% were rated as exaggerating the findings' importance and 26% of investigator quotes were considered to overstate the research importance [13]. Recently, a study showed that the quality of press releases influenced subsequent media coverage content [24].

Of course, press releases are not meant to be condensed versions of scientific papers; they are meant to summarize the most important findings, contextualize these finding for journalists, and provide contact details for authors and quotes. By being condensed, they always lack details that are contained in the papers. The use of “spin” or a particular emphasis could be a way to increase the interest of journalists and subsequent citations in the peer-reviewed literature.

However, this situation becomes problematic if it modifies readers' interpretation of research findings. Our results add to these previous studies by showing the link between the distorted presentation and interpretation of the results in scientific articles and the distorted content and interpretation of press releases. These findings raise the issue of the quality of the peer review process and highlight the importance of this process for disseminating accurate research results.

Our study has several limitations. Firstly, our sample included only published reports of RCTs with a press release indexed in the Eurekalert! database within a 4 mo period, and reported in English; this sample may not be representative of all press releases of RCT results. In fact, half of the press releases selected were written by press officers of medical journals with a high impact factor. Other sources of press releases exist on industry websites, medical journal websites, or other databases for journalists. However, the Eurekalert! database is one of the most important sources of freely available press releases, and most research published on press releases has used this database. Further, there is no reason to believe that the selection of the sample over only 4 mo would bias the results. Secondly, RCTs represent only a small part of the medical literature and the findings may not apply to media reporting of medical or scientific research as a whole. Thirdly, we searched for “spin” only in the article abstract conclusions, not in the entire published article. Consequently, we are not able to determine whether “spin” in the press release was the same as the “spin” in the whole article. We chose the abstract conclusions because it is the most accessible section of an article. Readers often base their initial assessment of a trial on the information reported in an abstract conclusion, and in some geographic areas, the abstract of an RCT report may be all that health professionals have easy access to [25],[26]. Fourthly, the content analysis and the interpretation coding were subjective [27]. However, two independent reviewers performed this assessment with consensus. Fifthly, we focused on articles and press releases of RCT results. We did not evaluate press releases for other study designs or proceedings of conferences.

In conclusion, previous work showed that exaggerated and inappropriate coverage of research findings in the news media is linked to inappropriate reporting of press releases. Our study adds to these results showing that “spin” in press releases and the news is related to the presence of “spin” in the published article, namely the abstract conclusions. Additionally, our work highlights that this inappropriate reporting could bias readers' interpretation of research results.

Consequently, reviewers and editors of published articles have an important role to play in the dissemination of research findings and should be particularly aware of the need to ensure that the conclusions reported are an appropriate reflection of the trial findings and do not overinterpret or misinterpret the results.

## Supporting Information

Text S1 Data abstraction form.
(DOC)

Text S2 Details related to the method.
(DOC)

Text S3 Kappa coefficient or agreement percentage for the assessment of “spin” in press releases and in articles.
(DOC)

Text S4 List of press releases and published articles examined.
(DOC)

Text S5 Multivariate analysis of factors associated with “spin” in press releases.
(DOC)

Text S6 Bivariate analysis of factors associated with “spin” in news items (*n* = 41).
(DOC)

Text S7 Bivariate analysis of factors associated with an overestimation of the benefit of the experimental treatment from the news as compared with the interpretation from the article abstract conclusions (*n* = 41).
(DOC)

## Abbreviations

RCT: randomized controlled trial

## References

[1] Schwartz LM, Woloshin S (2004) The media matter: a call for straightforward medical reporting. Ann Intern Med 140: 226–228. 14757622 10.7326/0003-4819-140-3-200402030-00015

[2] Jaques H (2011) BMJ Careers - get your research reported well in the news. Available: http://careers.bmj.com/careers/advice/view-article.html?id=20001805. Accessed 8 August 2012.

[3] Incomplete reporting of research in academic press releases. Lancet 373: 1920. 10.1016/S0140-6736(09)61044-8 19501726

[4] Woloshin S, Schwartz LM (2002) Press releases: translating research into news. JAMA 287: 2856–2858. 12038933 10.1001/jama.287.21.2856

[5] van Trigt AM, de Jong-van den Berg LT, Haaijer-Ruskamp FM, Willems J, Tromp TF (1994) Journalists and their sources of ideas and information on medicines. Soc Sci Med 38: 637–643. 8184327 10.1016/0277-9536(94)90261-5

[6] Kuriya B, Schneid EC, Bell CM (2008) Quality of pharmaceutical industry press releases based on original research. PLoS One 3: e2828 doi:10.1371/journal.pone.0002828. 18716675 10.1371/journal.pone.0002828 PMC2518517

[7] Chapman S, Nguyen TN, White C (2007) Press-released papers are more downloaded and cited. Tob Control 16: 71. 17297082 10.1136/tc.2006.019034 PMC2598438

[8] Entwistle V (1995) Reporting research in medical journals and newspapers. BMJ 310: 920–923. 7719187 10.1136/bmj.310.6984.920 PMC2549297

[9] Woloshin S, Schwartz LM, Kramer BS (2009) Promoting healthy skepticism in the news: helping journalists get it right. J Natl Cancer Inst 101: 1596–1599. 19933445 10.1093/jnci/djp409 PMC2786919

[10] Sackett DL, Rosenberg WM, Gray JA, Haynes RB, Richardson WS (1996) Evidence based medicine: what it is and what it isn't. BMJ 312: 71–72. 8555924 10.1136/bmj.312.7023.71 PMC2349778

[11] Phillips DP, Kanter EJ, Bednarczyk B, Tastad PL (1991) Importance of the lay press in the transmission of medical knowledge to the scientific community. N Engl J Med 325: 1180–1183. 1891034 10.1056/NEJM199110173251620

[12] Boutron I, Dutton S, Ravaud P, Altman DG (2010) Reporting and interpretation of randomized controlled trials with statistically nonsignificant results for primary outcomes. JAMA 303: 2058–2064. 20501928 10.1001/jama.2010.651

[13] Woloshin S, Schwartz LM, Casella SL, Kennedy AT, Larson RJ (2009) Press releases by academic medical centers: not so academic? Ann Intern Med 150: 613–618. 19414840 10.7326/0003-4819-150-9-200905050-00007

[14] Als-Nielsen B, Chen W, Gluud C, Kjaergard LL (2003) Association of funding and conclusions in randomized drug trials: a reflection of treatment effect or adverse events? JAMA 290: 921–928. 12928469 10.1001/jama.290.7.921

[15] Schwartz LM, Woloshin S (2002) News media coverage of screening mammography for women in their 40 s and tamoxifen for primary prevention of breast cancer. JAMA 287: 3136–3142. 12069679 10.1001/jama.287.23.3136

[16] Zou G (2004) A modified poisson regression approach to prospective studies with binary data. Am J Epidemiol 159: 702–706. 15033648 10.1093/aje/kwh090

[17] Austin PC, Tu JV (2004) Bootstrap methods for developing predictive models. The American Statistician 58: 131–137.

[18] Haas JS, Kaplan CP, Gerstenberger EP, Kerlikowske K (2004) Changes in the use of postmenopausal hormone therapy after the publication of clinical trial results. Ann Intern Med 140: 184–188. 14757616 10.7326/0003-4819-140-3-200402030-00009

[19] Grilli R, Ramsay C, Minozzi S (2002) Mass media interventions: effects on health services utilisation. Cochrane Database Syst Rev CD000389. 11869574 10.1002/14651858.CD000389 PMC13429285

[20] Sharma V, Dowd MD, Swanson DS, Slaughter AJ, Simon SD (2003) Influence of the news media on diagnostic testing in the emergency department. Arch Pediatr Adolesc Med 157: 257–260. 12622675 10.1001/archpedi.157.3.257

[21] Schwartz LM, Woloshin S, Welch HG (1999) Misunderstandings about the effects of race and sex on physicians' referrals for cardiac catheterization. N Engl J Med 341: 277–279. 10413743 10.1056/NEJM199907223410411

[22] Moynihan R, Bero L, Ross-Degnan D, Henry D, Lee K, et al (2000) Coverage by the news media of the benefits and risks of medications. N Engl J Med 342: 1645–1650. 10833211 10.1056/NEJM200006013422206

[23] Naylor CD, Chen E, Strauss B (1992) Measured enthusiasm: does the method of reporting trial results alter perceptions of therapeutic effectiveness? Ann Intern Med 117: 916–921. 1443954 10.7326/0003-4819-117-11-916

[24] Schwartz LM, Woloshin S, Andrews A, Stukel TA (2012) Influence of medical journal press releases on the quality of associated newspaper coverage: retrospective cohort study. BMJ 344: d8164. 22286507 10.1136/bmj.d8164 PMC3267473

[25] Hopewell S, Clarke M, Moher D, Wager E, Middleton P, et al (2008) CONSORT for reporting randomized controlled trials in journal and conference abstracts: explanation and elaboration. PLoS Med 5: e20 doi:10.1371/journal.pmed.0050020. 18215107 10.1371/journal.pmed.0050020 PMC2211558

[26] Gøtzsche PC (2006) Believability of relative risks and odds ratios in abstracts: cross sectional study. BMJ 333: 231–234. 16854948 10.1136/bmj.38895.410451.79 PMC1523498

[27] Horton R (2002) The hidden research paper. JAMA 287: 2775–2778. 12038909 10.1001/jama.287.21.2775

