# Supplementary material for: Misrepresentation of Randomized Controlled Trials in Press Releases and News Coverage: A Cohort Study
Source: PLoS Med. 2012 Sep 11;9(9):e1001308. doi: 10.1371/journal.pmed.1001308 (PMC3439420; doi:10.1371/journal.pmed.1001308)
Supplement: Text S1 — Data abstraction form. (DOC) [file pmed.1001308.s001.doc]

**Text S1. Data abstraction form**

**INTERPRETATION OF PRESS RELEASES**

N° PR: ……………

Initials reader:

**INTERPRETATION OF TITLE:**

1. Does the title leave you with the impression that all or some patients should :

Definitely obtain the experimental treatment evaluated

Probably obtain the experimental treatment evaluated

Patients should decide for themselves (i.e., the press release was neutral)

Probably not obtain the experimental treatment evaluated

Definitely not obtain the experimental treatment evaluated

Can’t conclude

1. Does the title leave you with the impression that there is

A lot

A little

No uncertainty about the benefit (or lack of benefit) of the experimental treatment evaluated

**INTERPRETATION OF PRESS RELEASE FULL TEXT:**

1. Does this press release leave you with the impression that all or some patients should :

Definitely obtain the experimental treatment evaluated

Probably obtain the experimental treatment evaluated

Patients should decide for themselves (i.e., the press release was neutral)

Probably not obtain the experimental treatment evaluated

Definitely not get the experimental treatment evaluated

1. Does this press release leave you with the impression that there is

A lot

A little

No uncertainty about the benefit (or lack of benefit) of the experimental treatment evaluated

Fiche de recueil

Reader ID : IB AY

Article ID: ……..

**ORIGINAL ARTICLE**

**GENERAL CHARACTERISTICS**

1. Publication journal:  General journal

Specialized journal

Impact factor: ……………………….

NEJM  JAMA Lancet Ann Int Med  BMJ  Plos med

1. Date of publication: (yyyy/mm)………………
2. Register number:  reported  not reported
3. Sample size: ……………..
4. Experimental treatment
   1. clearly identify:  Yes  No
   2. Experimental treatment:  Drug  surgery/procedure  device

Therapeutic strategy Participative  other

1. Comparator:  Placebo/attention control intervention  Usual care/ no treatment  Active treatment  Other Unclear
2. Funding source:  none  profit  non-profit

Both  not reported

1. Type of PO: Efficacy safety both unclear

**REPORTED RESULTS *(Results reported in the article: section methods and results)***

1. **Outcome**
   1. **Primary outcome(s)** [[1]](#footnote-2)
      1. clearly identified[[2]](#footnote-3) Yes No
      2. If yes, number of primary outcomes: ……….
      3. Results of primary outcome: All + All - some + / some – NR

*+ means p<0.05, - means p≥0.05*

- - 1. Primary outcome reported per arm: Yes with precision Yes without precision

No

*Precision= [IC] or (+/- sd)*

- - 1. At least one primary outcome reported with effect size: Yes with precision Yes without precision No

*Precision = OR, RR, RA, HR, means difference*

- 1. **Secondary outcomes**:

Results of secondary outcome: All + All - some + / some – NR

*+ means p<0.05, - means p≥05*

- 1. **Subgroup comparison:** Yes and adequate (pre-specified, interaction test, on primary outcome)  Yes and Inadequate  No
     1. If inadequate: not reported as pre-specified not on primary outcome no interaction test
  2. **Within (or overall within-group) group comparison:** Yes No
  3. **Adverse events reported**: yes per arms yes but not per arms NR
  4. **Exploratory analyzes** on another objective (i.e. not related to the treatment) YesNo

………………………………………………………………………………………

- 1. **Other:** Yes:No

**Title**

- 1. The title focused on efficacy safety no specific focus

Lack of efficacy  lack of safety *other*

- 1. What is the title content: no result

Focus on PO only

Focus on PO and other results

Focus on other results only

Other………………………………

- 1. There is spin[[3]](#footnote-4) in the title?  Yes No

If yes, describe……………………………………..

- 1. Is the title hype *(publicite/* inadequate emphasis) the treatment effect? Yes No
  2. Is there some caution (i.e. uncertainty) in the title  Yes No
  3. Other……………………………………………………………………..  Yes No

**Results reported in the abstract**

1. **Primary outcome clearly reported in the abstract** Yes  No
2. **Results reported In the abstract:**
   1. Significant primary outcome  Yes  No
   2. Nonsignificant primary outcome  Yes  No
   3. Significant secondary outcome  Yes  No
   4. Nonsignificant secondary outcome  Yes  No
   5. Significant subgroup results  Yes and adequate  Yes and inadequate No
   6. Nonsignificant subgroup results  Yes and adequate  Yes and inadequate No
   7. Positive within-group results  Yes  No
   8. Negative within-group results  Yes  No
   9. Exploratory analyses on another objective (i.e. not related to the treatment)YesNo
   10. Limitations  Yes  No
   11. Safety  Yes  No
   12. Other……………………………………….  Yes  No
3. **Spin in abstract conclusion**
   1. No acknowledgment of nonstatistically significant primary outcome Yes No
   2. Reporting nonstatistically significant results as if they

were statistically significant Yes No

- 1. Claiming equivalence when failure to demonstrate a difference Yes  No
  2. Focus on positive secondary outcome  Yes No
  3. Focus on inappropriate subgroup Yes No
  4. Focus on within group Yes No
  5. Negative outcome reported with linguistic spin:  Yes  No
  6. Focus on post hoc analyses  Yes  No
  7. Deviation from intent-to-treat analyses  Yes  No
  8. Ignore data on side effects  Yes  No
  9. Inadequate claim of safety  Yes  No
  10. Inappropriate extrapolation (i.e.: extension to other indications) Yes No
  11. Other spin………………………………………………………………………………  Yes  No

1. **Reports perspective** (section discussion)

Recommendations to use the treatment in clinical practice

Recommendations for further trial

No perspective

Other

1. **Is the conclusion written with uncertainty?:**  Yes No

**PRESS RELEASE**

**GENERAL CHARACTERISTICS**

1. Date of PR’s publication Dec09 Jan10 Feb10 Mar10
2. Origin of PR: Editor Industry Investigator institution
3. Does the PR facilitate access to full article:

Yes with electronic link to article

Yes with full reference

No (no link, no complete reference citation)

1. Contact with authors : Yes, authors Yes, press service Yes other No
2. Funding source reported: Yes No
3. Reporting of study design: yes no
4. Sample size reported: yes no
5. Reporting of length of follow up (for PO): yes no
6. Experimental treatment reported: yes no inadequately
7. Comparator reported: yes no inadequately
8. **Patient’s characteristics:**
   1. Inclusion criteria: mentioned not mentioned
   2. Exclusion criteria: mentioned not mentioned

**REPORTED RESULTS**

1. **Results reported**
   1. Significant primary outcome  Yes  No
   2. Nonsignificant primary outcome  Yes  No
   3. Significant secondary outcome  Yes  No
   4. Nonsignificant secondary outcome  Yes  No
   5. Significant subgroup results  Yes and adequate  Yes and InadequateNo
   6. Nonsignificant subgroup results  Yes and adequate  Yes and inadequateNo
   7. Positive within-group results  Yes  No
   8. Negative within-group results  Yes  No
   9. Exploratory analyses on another objective (i.e. not related to the treatment YesNo
   10. Other……………………………………….  Yes  No
2. **How are primary outcome(s)/result mentioned?**
   1. Words only  Yes  No
   2. Primary outcome reported per arm: Yes with precision Yes without precision No

*Precision= [IC] or (+/- sd)*

- 1. Primary outcome reported with effect sizeYes with precision Yes without precisionNo

*Precision = OR, RR, RA, HR, means difference*

1. **Safety**
   1. Are side effect/harms mentioned: Yes No
   2. Harm quantified: Yes No
2. **Limits of the study**:  reported  not reported
3. **Extrapolation** (i.e.: extension to other indications)Yes No

**FORM**

1. **Title**
   1. The title focused on efficacy safety no specific focus

Lack of efficacy  lack of safety *other*

- 1. What is the title content: no result

Focus on primary outcome only

Focus on primary outcome and other results Focus on other results only Other………………………………

- 1. There is spin in the title?  Yes No

if yes, describe……………………………………..

- 1. Is the title hype *(publicite/* inadequate emphasis) the treatment effect? YesNo
  2. Is there some caution (i.e. uncertainty) in the title  Yes No
  3. Other……………………………………………………………………..  Yes No

1. **Spin**
   1. No acknowledgment of nonstatistically significant primary outcome Yes No
   2. Reporting nonstatistically significant results as if they

were statistically significant Yes No

- 1. Claiming equivalence when failure to demonstrate a difference Yes  No
  2. Focus on positive secondary outcome  Yes No
  3. Focus on inappropriate subgroup Yes No
  4. Focus on within group Yes No
  5. Negative outcome reported with linguistic spin:  Yes No
  6. Focus on post hoc analyses  Yes  No
  7. Deviation from intent-to-treat analyses  Yes  No
  8. Ignore data on side effects  Yes  No
  9. Inadequate claim of safety  Yes  No
  10. Inappropriate extrapolation (i.e.: extension to other indications) Yes No
  11. Other spin………………………………………………………………………………  Yes  No

1. **Presentation**
   1. Interview or editorial:Yes by authors Yes by editorialist or expert No

**If yes, content of interviews:**

- - 1. neutral with results Yes No
    2. moderate the results (uncertainty, caution) Yes No
    3. affirmative on the results Yes No
    4. reported the results with emphasis Yes No
    5. other Yes No

…………………………………………………………………………………………………………

- 1. Take up from original article “ ” or “authors write”: Yes No

**If yes, content:**

- - 1. neutral with results Yes No
    2. moderate the results (uncertainty, caution) Yes No
    3. affirmative on the results Yes No
    4. reported the results with emphasis Yes No
    5. Other Yes No

………………………………………………………………………………………………………….

- 1. Other spin in the presentation Yes No

If yes, describe……………………………………………………………………………………………..

**INTERPRETATION OF PUBLISHED ARTICLE:**

1. Considering the trial results do you think patients should :

Definitely obtain the experimental treatment evaluated

Probably obtain the experimental treatment evaluated

Patients should decide for themselves (i.e., the article was neutral)

Probably not obtain the experimental treatment evaluated

Definitely not obtain the experimental treatment evaluated

1. Do the results leave you with the impression that there is :

A lot

A little

No uncertainty about the benefit (or lack of benefit) of the experimental treatment evaluated

1. When the primary outcome is not clearly identified, report all outcomes results as primary outcome and nothing in the secondary outcome section [↑](#footnote-ref-2)
2. Definition du PO (Chan, Lancet 2005): 1)primary or main defined explicitly, 2)outcome stated in power calculation, 3) described explicitly in primary study objectives , 4) if other = not clearly identified. [↑](#footnote-ref-3)
3. *Spin in title*: *title claiming treatment effectiveness or safety when primary outcome is negative, or title focus on treatment and disease with no statement of comparison or randomization or no question mark on the effect of treatment* [↑](#footnote-ref-4)
