# Supplementary material for: Misrepresentation of Randomized Controlled Trials in Press Releases and News Coverage: A Cohort Study
Source: PLoS Med. 2012 Sep 11;9(9):e1001308. doi: 10.1371/journal.pmed.1001308 (PMC3439420; doi:10.1371/journal.pmed.1001308)
Supplement: Text S2 — Details related to the method. (DOC) [file pmed.1001308.s002.doc]

**Text S2. Details related to the method**

**Assessment of published articles**

*General characteristics of RCTs*

For each selected article, the characteristics of the RCT were recorded: date of publication, journal (general versus specialized), impact factor, funding source (ie, for-profit, nonprofit or both, not reported, no funding), sample size, the experimental treatment (ie, drug, surgery or procedure, device, therapeutics strategy, participative or other) and comparator (placebo or attention control intervention, usual care or no treatment, active treatment, other or unclear).

We checked whether primary outcomes were clearly identified (ie, reported explicitly as such, stated in the power calculation, or described explicitly in primary study objectives). We recorded the number and type of primary outcomes (ie, efficacy, safety or both). From the results section of the full-text articles, including tables and figures, we recorded whether the results for primary outcome(s) were 1) all statistically significant, 2) all nonstatistically significant, 3) some statistically significant and some not, or 4) unclear. When the primary outcome was not clearly identified, all reported outcomes were considered the primary outcome(s). *Spin in the abstract conclusion of articles*

We defined “spin” as a specific reporting (intentional or unintentional) that emphasizes the beneficial effect of the experimental treatment. We read the full text article and then searched for “spin” in the abstract conclusions as a proxy of “spin” in the article. We focused on abstract conclusions because 1) the abstract is the freely accessible part of article, 2) readers often base their initial assessment of a trial’s results on the information reported in the abstract conclusions and 3) the abstract conclusions is the section where “spin” is the most prevalent. We used a classification of “spin” described in a previous work. This classification was initially developed in the context of trials with a nonstatistically significant primary outcome. This classification was adapted to all RCTs. We considered “spin” as being an inadequate focus on statistically significant results (within-group comparison, secondary outcomes, subgroup analyses, modified population of analyses); an interpretation of statistically nonsignificant results for the primary outcomes as showing treatment equivalence or comparable effectiveness; or any inadequate claim of safety or emphasis of the beneficial effect of the treatment.

**Assessment of press releases**

*General characteristics of press releases*

For each selected press release, we recorded the date of publication of the press release, the author of the press release (press officer, industry or marketing agency, investigator or institution), and whether the release provided easy access to the related published article (ie, electronic link or full reference). We recorded whether the main characteristics of the trial were reported (ie, funding, sample size, design, length of follow-up), whether an interview from an author of the trial report, independent expert or editorialist was included and whether the press release contained any quotation extracted from the original article. We recorded whether the results mentioned in the interview and quotations were reported with moderation, affirmation or emphasis. We assessed how the trial results were mentioned: in words only, quantified per arm with precision or not, or quantified with an effect size with precision or not.

*Spin in press releases*

We systematically searched for “spin” in the full text of the press release using the same classification.

**Assessment of the news**

We systematically searched for “spin” in the full text of the news and determined whether the spin was similar to the spin reported in the press release or was new spin not previously reported in the press release.
