# Supplementary material for: Misrepresentation of Randomized Controlled Trials in Press Releases and News Coverage: A Cohort Study
Source: PLoS Med. 2012 Sep 11;9(9):e1001308. doi: 10.1371/journal.pmed.1001308 (PMC3439420; doi:10.1371/journal.pmed.1001308)
Supplement: Text S3 — Kappa coefficient or agreement percentage for the assessment of “spin” in press releases and in articles. (DOC) [file pmed.1001308.s003.doc]

**Text S3**. Kappa coefficient or agreement percentage for the assessment of spin in press releases and in articles

| Type of spin | Spin in press releases | | Spin in published article s | |
| --- | --- | --- | --- | --- |
|  | Kappa [95% CI] | Agreement  % [95% CI] | Kappa [95% CI] | Agreement  % [95% CI] |
| No acknowledgment of non-statistically significant primary outcome | 0.95 [0.86-1.00] | 97% [0.90-1] | 0.77 [0.58-0.96] | 93% [0.84-0.98] |
| Reporting non-statistically significant results as if they were statistically significant | 0.47 [0.02-0.91] | 93% [0.84-0.98] | 0 | 96% [0.88-0.99] |
| Claiming equivalence when failure to demonstrate a difference | 0.92 [0.78-1.00] | 99% [0.92-1.00] | 0.78 [0.49-1.00] | 97% [0.90-1] |
| Focus on positive secondary outcome | 0.50 [0.13-0.88] | 90% [0.81-0.96] | 0.70 [0.4-1.00] | 96% [0.88-0.99] |
| Focus on inappropriate subgroup | 0.79 [0.40-1.00] | 96% [0.88-0.99] | 0.21 [-0.19-0.61] | 91% [0.82-0.97] |
| Focus on within group | 0.67[0.39-0.94] | 93% [0.84-0.98] | 0.26 [-018-0.70] | 93% [0.84-0.98] |
| Negative outcome reported with linguistic spin | 0.32 [-0.15-0.79] | 94% [0.86-0.98] | 0.79 [0.40-1.00] | 96% [0.88-0.99] |
| Focus on post-hoc analyses | 0 | 99% [0.92-1.00] | 0 | 98% [0.92-1.00] |
| Deviation from ITT analyses | . | 100% [0.96-1.00] | . | 100% [0.96-1.00] |
| Ignore data on safety | 0.55 [0.11-1.00] | 94% [0.86-0.98] | 0.32 [-0.15- 0.79] | 94% [0.86-0.98] |
| Inadequate claim of safety | 0.65 [0.28-1.00] | 96% [0.88-0.99] | 0.57 [0.20-0.96] | 94% [0.86-0.98] |
| Inappropriate extrapolation | 0.65 [0.20-1.00] | 96% [0.88-0.99] | 0.49 [-0.11-1.00] | 97% [0.90-1] |

95% CI, 95% confidence interval
