# Supplementary material for: Misrepresentation of Randomized Controlled Trials in Press Releases and News Coverage: A Cohort Study
Source: PLoS Med. 2012 Sep 11;9(9):e1001308. doi: 10.1371/journal.pmed.1001308 (PMC3439420; doi:10.1371/journal.pmed.1001308)
Supplement: Text S4 — List of press releases and published articles examined. (DOC) [file pmed.1001308.s004.doc]

Text S4. List of press releases and published articles examined

| **N** | **Title of press releases included** | **References of published articles included** |
| --- | --- | --- |
| 1 | [Fish oil not snake oil](http://ekasearch01.eurekalert.org/e3/cs.html?url=http%3A//www.eurekalert.org/pub_releases/2010-01/bc-fon011510.php&charset=iso-8859-1&qt=%2Brandom*%2C+%2Btype%3A"research"%2C+keywords%3A"Medicine/Health"%2C+EurekAlert&col=ev3rel&n=1&la=en) | Barbosa VM, Miles EA, Calhau C, Lafuente E, Calder PC. Effects of a fish oil containing lipid emulsion on plasma phospholipid fatty acids, inflammatory markers, and clinical outcomes in septic patients: a randomized, controlled clinical trial. Crit Care. 2010;14(1): |
| 2 | [Atrial fibrillation treatment with catheter shows better results than drug therapy](http://ekasearch01.eurekalert.org/e3/cs.html?url=http%3A//www.eurekalert.org/pub_releases/2010-01/jaaj-aft012110.php&charset=iso-8859-1&qt=%2Brandom*%2C+%2Btype%3A"research"%2C+keywords%3A"Medicine/Health"%2C+EurekAlert&col=ev3rel&n=3&la=en) | Wilber DJ, Pappone C, Neuzil P, De Paola A, Marchlinski F, Natale A, et al.  Comparison of antiarrhythmic drug therapy and radiofrequency catheter ablation in  patients with paroxysmal atrial fibrillation: a randomized controlled trial. JAMA. 2010  janv 27;303(4):333-340. |
| 3 | [Exercise associated with preventing, improving mild cognitive impairment](http://ekasearch01.eurekalert.org/e3/cs.html?url=http%3A//www.eurekalert.org/pub_releases/2010-01/jaaj-eaw010710.php&charset=iso-8859-1&qt=%2Brandom*%2C+%2Btype%3A"research"%2C+keywords%3A"Medicine/Health"%2C+EurekAlert&col=ev3rel&n=8&la=en) | Baker LD, Frank LL, Foster-Schubert K, Green PS, Wilkinson CW, McTiernan A, et al. Effects of aerobic exercise on mild cognitive impairment: a controlled trial. Arch. Neurol. 2010 janv;67(1):71-79. |
| 4 | [Vaccine approach extends life of metastatic prostate cancer patients](http://ekasearch01.eurekalert.org/e3/cs.html?url=http%3A//www.eurekalert.org/pub_releases/2010-01/dci-vae012210.php&charset=iso-8859-1&qt=%2Brandom*%2C+EurekAlert&col=ev3rel&n=16&la=en) | Kantoff PW, Schuetz TJ, Blumenstein BA, Glode LM, Bilhartz DL, Wyand M, et al. Overall survival analysis of a phase II randomized controlled trial of a Poxviral-based PSA-targeted immunotherapy in metastatic castration-resistant prostate cancer. J. Clin. Oncol. 2010 mars 1;28(7):1099-1105. |
| 5 | [New research shows it is possible to reduce antibiotic use in intensive care without ...](http://ekasearch01.eurekalert.org/e3/cs.html?url=http%3A//www.eurekalert.org/pub_releases/2010-01/l-nrs012110.php&charset=iso-8859-1&qt=%2Brandom*%2C+EurekAlert&col=ev3rel&n=18&la=en) | Bouadma L, Luyt C-E, Tubach F, Cracco C, Alvarez A, Schwebel C, et al. Use of procalcitonin to reduce patients’ exposure to antibiotics in intensive care units (PRORATA trial): a multicentre randomised controlled trial. Lancet. 2010 févr 6;375(9713):463-474. |
| 6 | [Does electro-acupuncture prevent prolonged postoperative ileus?](http://ekasearch01.eurekalert.org/e3/cs.html?url=http%3A//www.eurekalert.org/pub_releases/2010-01/wjog-dep011410.php&charset=iso-8859-1&qt=%2Brandom*%2C+EurekAlert&col=ev3rel&n=27&la=en) | Meng Z-Q, Garcia MK, Chiang JS, Peng H-T, Shi Y-Q, Fu J, et al. Electro-acupuncture to  prevent prolonged postoperative ileus: a randomized clinical trial. World J.  Gastroenterol. 2010 janv 7;16(1):104-111. |
| 7 | [Not sedating critically ill patients means they need fewer days on mechanical ventilation, ...](http://ekasearch01.eurekalert.org/e3/cs.html?url=http%3A//www.eurekalert.org/pub_releases/2010-01/l-nsc012710.php&charset=iso-8859-1&qt=%2Brandom*%2C+EurekAlert&col=ev3rel&n=44&la=en) | Strøm T, Martinussen T, Toft P. A protocol of no sedation for critically ill patients receiving mechanical ventilation: a randomised trial. Lancet. 2010 févr 6;375(9713):475-480. |
| 8 | [Lighter sedation for elderly during surgery may reduce risk of confusion, disorientation ...](http://ekasearch01.eurekalert.org/e3/cs.html?url=http%3A//www.eurekalert.org/pub_releases/2010-01/jhmi-lsf012110.php&charset=iso-8859-1&qt=%2Brandom*%2C+EurekAlert&col=ev3rel&n=57&la=en) | Sieber FE, Zakriya KJ, Gottschalk A, Blute M-R, Lee HB, Rosenberg PB, et al. Sedation depth during spinal anesthesia and the development of postoperative delirium in elderly patients undergoing hip fracture repair. Mayo Clin. Proc. 2010 janv;85(1):18-26 |
| 9 | [Herpes medication does not reduce risk of HIV transmission, UW-led international study ...](http://ekasearch01.eurekalert.org/e3/cs.html?url=http%3A//www.eurekalert.org/pub_releases/2010-01/uow--hmd012010.php&charset=iso-8859-1&qt=%2Brandom*%2C+EurekAlert&col=ev3rel&n=61&la=en) | Celum C, Wald A, Lingappa JR, Magaret AS, Wang RS, Mugo N, et al. Acyclovir and  transmission of HIV-1 from persons infected with HIV-1 and HSV-2. N. Engl. J. Med.  2010 févr 4;362(5):427-439. |
| 10 | [New treatment shown to reduce recurrence of debilitating diarrhea](http://ekasearch01.eurekalert.org/e3/cs.html?url=http%3A//www.eurekalert.org/pub_releases/2010-01/uomm-nts011910.php&charset=iso-8859-1&qt=%2Brandom*%2C+EurekAlert&col=ev3rel&n=62&la=en) | Lowy I, Molrine DC, Leav BA, Blair BM, Baxter R, Gerding DN, et al. Treatment with  monoclonal antibodies against Clostridium difficile toxins. N. Engl. J. Med. 2010 janv  21;362(3):197-205. |
| 11 | [HPV testing prevents more invasive cervical cancers than cytology](http://ekasearch01.eurekalert.org/e3/cs.html?url=http%3A//www.eurekalert.org/pub_releases/2010-01/l-htp011510.php&charset=iso-8859-1&qt=%2Brandom*%2C+EurekAlert&col=ev3rel&n=68&la=en) | Ronco G, Giorgi-Rossi P, Carozzi F, Confortini M, Dalla Palma P, Del Mistro A, et al.  Efficacy of human papillomavirus testing for the detection of invasive cervical cancers  and cervical intraepithelial neoplasia: a randomised controlled trial. Lancet Oncol.  2010 mars;11(3):249-257. |
| 12 | [Ticagrelor for heart attack patients -- a landmark event that should redefine patient care](http://ekasearch01.eurekalert.org/e3/cs.html?url=http%3A//www.eurekalert.org/pub_releases/2010-01/l-tfh011210.php&charset=iso-8859-1&qt=%2Brandom*%2C+EurekAlert&col=ev3rel&n=76&la=en) | Cannon CP, Harrington RA, James S, Ardissino D, Becker RC, Emanuelsson H, et al.  Comparison of ticagrelor with clopidogrel in patients with a planned invasive strategy  for acute coronary syndromes (PLATO): a randomised double-blind study. Lancet.  2010 janv 23;375(9711):283-293. |
| 13 | [Newer treatment for Achilles tendon disorder does not appear to be effective](http://ekasearch01.eurekalert.org/e3/cs.html?url=http%3A//www.eurekalert.org/pub_releases/2010-01/jaaj-ntf010710.php&charset=iso-8859-1&qt=%2Brandom*%2C+EurekAlert&col=ev3rel&n=80&la=en) | de Vos RJ, Weir A, van Schie HTM, Bierma-Zeinstra SMA, Verhaar JAN, Weinans H, et al. Platelet-rich plasma injection for chronic Achilles tendinopathy: a randomized controlled trial. JAMA. 2010 janv 13;303(2):144-149. |
| 14 | [Everolimus-eluting stent better than paclitaxel-eluting stent in unselected patients](http://ekasearch01.eurekalert.org/e3/cs.html?url=http%3A//www.eurekalert.org/pub_releases/2010-01/l-esb010610.php&charset=iso-8859-1&qt=%2Brandom*%2C+EurekAlert&col=ev3rel&n=89&la=en) | Kedhi E, Joesoef KS, McFadden E, Wassing J, van Mieghem C, Goedhart D, et al. Second-generation everolimus-eluting and paclitaxel-eluting stents in real-life practice (COMPARE): a randomised trial. Lancet. 2010 janv 16;375(9710):201-209. |
| 15 | [Immune responses to tetanus vaccine unchanged for RA patients on rituximab](http://ekasearch01.eurekalert.org/e3/cs.html?url=http%3A//www.eurekalert.org/pub_releases/2010-01/w-irt010610.php&charset=iso-8859-1&qt=%2Brandom*%2C+EurekAlert&col=ev3rel&n=96&la=en) | Bingham CO 3rd, Looney RJ, Deodhar A, Halsey N, Greenwald M, Codding C, et al.  Immunization responses in rheumatoid arthritis patients treated with rituximab:  results from a controlled clinical trial. Arthritis Rheum. 2010 janv;62(1):64-74. |
| 16 | [St. John's wort not helpful treatment for irritable bowel syndrome, Mayo Clinic researchers ...](http://ekasearch01.eurekalert.org/e3/cs.html?url=http%3A//www.eurekalert.org/pub_releases/2010-01/mc-sjw010410.php&charset=iso-8859-1&qt=%2Brandom*%2C+EurekAlert&col=ev3rel&n=105&la=en) | Saito YA, Rey E, Almazar-Elder AE, Harmsen WS, Zinsmeister AR, Locke GR, et al. A randomized, double-blind, placebo-controlled trial of St John’s wort for treating irritable bowel syndrome. Am. J. Gastroenterol. 2010 janv;105(1):170-177. |
| 17 | [Acupuncture reduces hot flashes, improves sex drive for breast cancer patients](http://ekasearch01.eurekalert.org/e3/cs.html?url=http%3A//www.eurekalert.org/pub_releases/2009-12/hfhs-arh122909.php&charset=iso-8859-1&qt=%2Brandom*%2C+%2Btype%3A"research"%2C+keywords%3A"Medicine/Health"%2C+EurekAlert&col=ev3rel&n=4&la=en) | Walker EM, Rodriguez AI, Kohn B, Ball RM, Pegg J, Pocock JR, et al. Acupuncture versus venlafaxine for the management of vasomotor symptoms in patients with hormone receptor-positive breast cancer: a randomized controlled trial. J. Clin. Oncol. 2010 févr 1;28(4):634-640. |
| 18 | [Reducing TV time helps adults burn more calories](http://ekasearch01.eurekalert.org/e3/cs.html?url=http%3A//www.eurekalert.org/pub_releases/2009-12/jaaj-rtt121009.php&charset=iso-8859-1&qt=%2Brandom*%2C+%2Btype%3A"research"%2C+keywords%3A"Medicine/Health"%2C+EurekAlert&col=ev3rel&n=6&la=en) | Otten JJ, Jones KE, Littenberg B, Harvey-Berino J. Effects of television viewing reduction on energy intake and expenditure in overweight and obese adults: a randomized controlled trial. Arch. Intern. Med. 2009 déc 14;169(22):2109-2115 |
| 19 | [Weight-loss proves effective cure for sleep apnea](http://ekasearch01.eurekalert.org/e3/cs.html?url=http%3A//www.eurekalert.org/pub_releases/2009-12/ki-wp120409.php&charset=iso-8859-1&qt=%2Brandom*%2C+%2Btype%3A"research"%2C+keywords%3A"Medicine/Health"%2C+EurekAlert&col=ev3rel&n=10&la=en) | Johansson K, Neovius M, Lagerros YT, Harlid R, Rössner S, Granath F, et al. Effect of a very low energy diet on moderate and severe obstructive sleep apnoea in obese men: a randomised controlled trial. BMJ. 2009;339:b4609. |
| 20 | [Breathlessness eased in patients with rare, often fatal disease](http://ekasearch01.eurekalert.org/e3/cs.html?url=http%3A//www.eurekalert.org/pub_releases/2009-12/uorm-bei121609.php&charset=iso-8859-1&qt=%2Brandom*%2C+EurekAlert&col=ev3rel&n=19&la=en) | Hiremath J, Thanikachalam S, Parikh K, Shanmugasundaram S, Bangera S, Shapiro L, et al. Exercise improvement and plasma biomarker changes with intravenous treprostinil therapy for pulmonary arterial hypertension: a placebo-controlled trial. J. Heart Lung Transplant. 2010 févr;29(2):137-149. |
| 21 | [Drug for Alzheimer's disease does not appear to slow cognitive decline](http://ekasearch01.eurekalert.org/e3/cs.html?url=http%3A//www.eurekalert.org/pub_releases/2009-12/jaaj-dfa121009.php&charset=iso-8859-1&qt=%2Brandom*%2C+EurekAlert&col=ev3rel&n=23&la=en) | Green RC, Schneider LS, Amato DA, Beelen AP, Wilcock G, Swabb EA, et al. Effect of tarenflurbil on cognitive decline and activities of daily living in patients with mild Alzheimer disease: a randomized controlled trial. JAMA. 2009 déc 16;302(23):2557-2564 |
| 22 | [Anemia drug not helpful for kidney disease patients](http://ekasearch01.eurekalert.org/e3/cs.html?url=http%3A//www.eurekalert.org/pub_releases/2009-12/usmc-adn122109.php&charset=iso-8859-1&qt=%2Brandom*%2C+EurekAlert&col=ev3rel&n=40&la=en) | Pfeffer MA, Burdmann EA, Chen C-Y, Cooper ME, de Zeeuw D, Eckardt K-U, et al. A trial of darbepoetin alfa in type 2 diabetes and chronic kidney disease. N. Engl. J. Med. 2009 nov 19;361(21):2019-2032. |
| 23 | [Good cholesterol not as protective in people with type 2 diabetes](http://ekasearch01.eurekalert.org/e3/cs.html?url=http%3A//www.eurekalert.org/pub_releases/2009-12/aha-gcn122109.php&charset=iso-8859-1&qt=%2Brandom*%2C+EurekAlert&col=ev3rel&n=43&la=en) | Sorrentino SA, Besler C, Rohrer L, Meyer M, Heinrich K, Bahlmann FH, et al.  Endothelial-vasoprotective effects of high-density lipoprotein are impaired in patients  with type 2 diabetes mellitus but are improved after extended-release niacin therapy.  Circulation. 2010 janv 5;121(1):110-122. |
| 24 | [1 dose of H1N1 vaccine may provide sufficient protection for infants and children](http://ekasearch01.eurekalert.org/e3/cs.html?url=http%3A//www.eurekalert.org/pub_releases/2009-12/jaaj-odo121709.php&charset=iso-8859-1&qt=%2Brandom*%2C+EurekAlert&col=ev3rel&n=45&la=en) | Nolan T, McVernon J, Skeljo M, Richmond P, Wadia U, Lambert S, et al. Immunogenicity of a monovalent 2009 influenza A(H1N1) vaccine in infants and children: a randomized trial. JAMA. 2010 janv 6;303(1):37-46. |
| 25 | [Gefitinib improves survival compared with standard chemotherapy in lung cancer patients ...](http://ekasearch01.eurekalert.org/e3/cs.html?url=http%3A//www.eurekalert.org/pub_releases/2009-12/l-gis121709.php&charset=iso-8859-1&qt=%2Brandom*%2C+EurekAlert&col=ev3rel&n=46&la=en) | Mitsudomi T, Morita S, Yatabe Y, Negoro S, Okamoto I, Tsurutani J, et al. Gefitinib versus cisplatin plus docetaxel in patients with non-small-cell lung cancer harbouring mutations of the epidermal growth factor receptor (WJTOG3405): an open label, randomised phase 3 trial. Lancet Oncol. 2010 févr;11(2):121-128 |
| 26 | [Psychotherapy offers obesity prevention for 'at risk' teenage girls](http://ekasearch01.eurekalert.org/e3/cs.html?url=http%3A//www.eurekalert.org/pub_releases/2009-12/w-poo121509.php&charset=iso-8859-1&qt=%2Brandom*%2C+EurekAlert&col=ev3rel&n=57&la=en) | Tanofsky-Kraff M, Wilfley DE, Young JF, Mufson L, Yanovski SZ, Glasofer DR, et al. A  pilot study of interpersonal psychotherapy for preventing excess weight gain in  adolescent girls at-risk for obesity. Int J Eat Disord. 2010 déc;43(8):701-706. |
| 27 | [Moderate weight loss in obese people improves heart function](http://ekasearch01.eurekalert.org/e3/cs.html?url=http%3A//www.eurekalert.org/pub_releases/2009-12/wuso-mwl120809.php&charset=iso-8859-1&qt=%2Brandom*%2C+EurekAlert&col=ev3rel&n=73&la=en) | de las Fuentes L, Waggoner AD, Mohammed BS, Stein RI, Miller BV 3rd, Foster GD, et al. Effect of moderate diet-induced weight loss and weight regain on cardiovascular structure and function. J. Am. Coll. Cardiol. 2009 déc 15;54(25):2376-2381. |
| 28 | [Treating cluster headaches with high-flow oxygen appears effective](http://ekasearch01.eurekalert.org/e3/cs.html?url=http%3A//www.eurekalert.org/pub_releases/2009-12/jaaj-tch120309.php&charset=iso-8859-1&qt=%2Brandom*%2C+EurekAlert&col=ev3rel&n=85&la=en) | Cohen AS, Burns B, Goadsby PJ. High-flow oxygen for treatment of cluster headache: a randomized trial. JAMA. 2009 déc 9;302(22):2451-2457. |
| 29 | [FDA-approved drug may slow beta cell destruction in type 1 diabetes patients](http://ekasearch01.eurekalert.org/e3/cs.html?url=http%3A//www.eurekalert.org/pub_releases/2009-12/usmc-fdm120409.php&charset=iso-8859-1&qt=%2Brandom*%2C+EurekAlert&col=ev3rel&n=97&la=en) | Pescovitz MD, Greenbaum CJ, Krause-Steinrauf H, Becker DJ, Gitelman SE, Goland R, et al. Rituximab, B-lymphocyte depletion, and preservation of beta-cell function. N. Engl. J. Med. 2009 nov 26;361(22):2143-2152. |
| 30 | [Smoking cessation results mixed among Ohio's Appalachian women](http://ekasearch01.eurekalert.org/e3/cs.html?url=http%3A//www.eurekalert.org/pub_releases/2009-12/aafc-scr113009.php&charset=iso-8859-1&qt=%2Brandom*%2C+EurekAlert&col=ev3rel&n=101&la=en) | Wewers ME, Ferketich AK, Harness J, Paskett ED. Effectiveness of a nurse-managed, lay-led tobacco cessation intervention among ohio appalachian women. Cancer Epidemiol. Biomarkers Prev. 2009 déc;18(12):3451-3458. |
| 31 | [Videos can help cancer patients choose level of care they prefer](http://ekasearch01.eurekalert.org/e3/cs.html?url=http%3A//www.eurekalert.org/pub_releases/2009-12/mgh-vch120209.php&charset=iso-8859-1&qt=%2Brandom*%2C+EurekAlert&col=ev3rel&n=109&la=en) | El-Jawahri A, Podgurski LM, Eichler AF, Plotkin SR, Temel JS, Mitchell SL, et al. Use of video to facilitate end-of-life discussions with patients with cancer: a randomized controlled trial. J. Clin. Oncol. 2010 janv 10;28(2):305-310 |
| 32 | [Study shows pine bark improves circulation, swelling and visual acuity in early diabetic ...](http://ekasearch01.eurekalert.org/e3/cs.html?url=http%3A//www.eurekalert.org/pub_releases/2009-12/mg-ssp113009.php&charset=iso-8859-1&qt=%2Brandom*%2C+EurekAlert&col=ev3rel&n=110&la=en) | Steigerwalt R, Belcaro G, Cesarone MR, Di Renzo A, Grossi MG, Ricci A, et al. Pycnogenol improves microcirculation, retinal edema, and visual acuity in early diabetic retinopathy. J Ocul Pharmacol Ther. 2009 déc;25(6):537-540. |
| 33 | [Addition of MRI to conventional assessment for breast cancer diagnosis has no effect on …](http://ekasearch01.eurekalert.org/e3/cs.html?url=http%3A//www.eurekalert.org/pub_releases/2010-02/l-aom021010.php&charset=iso-8859-1&qt=%2Brandom*%2C+%2Btype%3A"research"%2C+keywords%3A"Medicine/Health"%2C+EurekAlert&col=ev3rel&n=1&la=en) | Turnbull L, Brown S, Harvey I, Olivier C, Drew P, Napp V, et al. Comparative effectiveness of MRI in breast cancer (COMICE) trial: a randomised controlled trial. Lancet. 2010 févr 13;375(9714):563-571. |
| 34 | [Prednisolone not beneficial in most cases of community-acquired pneumonia](http://ekasearch01.eurekalert.org/e3/cs.html?url=http%3A//www.eurekalert.org/pub_releases/2010-02/ats-pnb022310.php&charset=iso-8859-1&qt=%2Brandom*%2C+%2Btype%3A"research"%2C+keywords%3A"Medicine/Health"%2C+EurekAlert&col=ev3rel&n=5&la=en) | Snijders D, Daniels JMA, de Graaff CS, van der Werf TS, Boersma WG. Efficacy of corticosteroids in community-acquired pneumonia: a randomized double-blinded clinical trial. Am. J. Respir. Crit. Care Med. 2010 mai 1;181(9):975-982. |
| 35 | [No difference in survival between combination and single therapy in renal cell carcinoma](http://ekasearch01.eurekalert.org/e3/cs.html?url=http%3A//www.eurekalert.org/pub_releases/2010-02/l-ndi020910.php&charset=iso-8859-1&qt=%2Brandom*%2C+EurekAlert&col=ev3rel&n=12&la=en) | Gore ME, Griffin CL, Hancock B, Patel PM, Pyle L, Aitchison M, et al. Interferon alfa-2a versus combination therapy with interferon alfa-2a, interleukin-2, and fluorouracil in patients with untreated metastatic renal cell carcinoma (MRC RE04/EORTC GU 30012): an open-label randomised trial. Lancet. 2010 févr 20;375(9715):641-648. |
| 36 | [Antimicrobial treatment for buruli ulcer is effective in early, limited disease](http://ekasearch01.eurekalert.org/e3/cs.html?url=http%3A//www.eurekalert.org/pub_releases/2010-02/l-atf020210.php&charset=iso-8859-1&qt=%2Brandom*%2C+EurekAlert&col=ev3rel&n=15&la=en) | Nienhuis WA, Stienstra Y, Thompson WA, Awuah PC, Abass KM, Tuah W, et al. Antimicrobial treatment for early, limited Mycobacterium ulcerans infection: a randomised controlled trial. Lancet. 2010 févr 20;375(9715):664-672. |
| 37 | [Combined drug therapy to treat TB and HIV significantly improves survival](http://ekasearch01.eurekalert.org/e3/cs.html?url=http%3A//www.eurekalert.org/pub_releases/2010-02/cums-cdt022410.php&charset=iso-8859-1&qt=%2Brandom*%2C+EurekAlert&col=ev3rel&n=17&la=en) | Abdool Karim SS, Naidoo K, Grobler A, Padayatchi N, Baxter C, Gray A, et al. Timing of  initiation of antiretroviral drugs during tuberculosis therapy. N. Engl. J. Med. 2010  févr 25;362(8):697-706. |
| 38 | [Study supports alternative anti-seizure medication following acute brain injury](http://ekasearch01.eurekalert.org/e3/cs.html?url=http%3A//www.eurekalert.org/pub_releases/2010-02/uoca-ssa021710.php&charset=iso-8859-1&qt=%2Brandom*%2C+EurekAlert&col=ev3rel&n=29&la=en) | Szaflarski JP, Sangha KS, Lindsell CJ, Shutter LA. Prospective, randomized, single-blinded comparative trial of intravenous levetiracetam versus phenytoin for seizure prophylaxis. Neurocrit Care. 2010 avr;12(2):165-172. |
| 39 | [Obese teens who receive gastric banding achieve significant weight loss](http://ekasearch01.eurekalert.org/e3/cs.html?url=http%3A//www.eurekalert.org/pub_releases/2010-02/jaaj-otw020410.php&charset=iso-8859-1&qt=%2Brandom*%2C+EurekAlert&col=ev3rel&n=42&la=en) | O’Brien PE, Sawyer SM, Laurie C, Brown WA, Skinner S, Veit F, et al. Laparoscopic adjustable gastric banding in severely obese adolescents: a randomized trial. JAMA. 2010 févr 10;303(6):519-526 |
| 40 | [Flower power can still calm the masses](http://ekasearch01.eurekalert.org/e3/cs.html?url=http%3A//www.eurekalert.org/pub_releases/2010-02/fo1b-fpc020810.php&charset=iso-8859-1&qt=%2Brandom*%2C+EurekAlert&col=ev3rel&n=43&la=en) | Amsterdam JD, Li Y, Soeller I, Rockwell K, Mao JJ, Shults J. A randomized, double-blind, placebo-controlled trial of oral Matricaria recutita (chamomile) extract therapy for generalized anxiety disorder. J Clin Psychopharmacol. 2009 août;29(4):378-382 |
| 41 | [Hypnosis can help control pain among women with metastatic breast cancer, UB Researcher ...](http://ekasearch01.eurekalert.org/e3/cs.html?url=http%3A//www.eurekalert.org/pub_releases/2010-02/uab-hch022610.php&charset=iso-8859-1&qt=%2Brandom*%2C+EurekAlert&col=ev3rel&n=49&la=en) | Butler LD, Koopman C, Neri E, Giese-Davis J, Palesh O, Thorne-Yocam KA, et al. Effects of supportive-expressive group therapy on pain in women with metastatic breast cancer. Health Psychol. 2009 sept;28(5):579-587 |
| 42 | [New intervention helps Latino parents of asthmatic children quit smoking](http://ekasearch01.eurekalert.org/e3/cs.html?url=http%3A//www.eurekalert.org/pub_releases/2010-02/l-nih021710.php&charset=iso-8859-1&qt=%2Brandom*%2C+EurekAlert&col=ev3rel&n=77&la=en) | Borrelli B, McQuaid EL, Novak SP, Hammond SK, Becker B. Motivating Latino caregivers of children with asthma to quit smoking: a randomized trial. J Consult Clin Psychol. 2010 févr;78(1):34-43. |
| 43 | [Walking linked to eased osteoarthritis](http://ekasearch01.eurekalert.org/e3/cs.html?url=http%3A//www.eurekalert.org/pub_releases/2010-02/bc-wlt021510.php&charset=iso-8859-1&qt=%2Brandom*%2C+EurekAlert&col=ev3rel&n=84&la=en) | Ng NTM, Heesch KC, Brown WJ. Efficacy of a progressive walking program and glucosamine sulphate supplementation on osteoarthritic symptoms of the hip and knee: a feasibility trial. Arthritis Res. Ther. 2010;12(1):R25 |
| 44 | [WHI data confirm short-term heart disease risks of combination menopausal hormone therapy](http://ekasearch01.eurekalert.org/e3/cs.html?url=http%3A//www.eurekalert.org/pub_releases/2010-02/nhla-wdc021510.php&charset=iso-8859-1&qt=%2Brandom*%2C+EurekAlert&col=ev3rel&n=89&la=en) | Toh S, Hernández-Díaz S, Logan R, Rossouw JE, Hernán MA. Coronary heart disease in postmenopausal recipients of estrogen plus progestin therapy: does the increased risk ever disappear? A randomized trial. Ann. Intern. Med. 2010 févr 16;152(4):211-217. |
| 45 | [Treatment for herpes in patients co-infected with herpes and HIV could delay HIV disease ...](http://ekasearch01.eurekalert.org/e3/cs.html?url=http%3A//www.eurekalert.org/pub_releases/2010-02/l-tfh021110.php&charset=iso-8859-1&qt=%2Brandom*%2C+EurekAlert&col=ev3rel&n=90&la=en) | Lingappa JR, Baeten JM, Wald A, Hughes JP, Thomas KK, Mujugira A, et al. Daily acyclovir for HIV-1 disease progression in people dually infected with HIV-1 and herpes simplex virus type 2: a randomised placebo-controlled trial. Lancet. 2010 mars 6;375(9717):824-833. |
| 46 | [High-altitude climbs may cause corneal swelling, but do not appear to affect vision](http://ekasearch01.eurekalert.org/e3/cs.html?url=http%3A//www.eurekalert.org/pub_releases/2010-02/jaaj-hcm020410.php&charset=iso-8859-1&qt=%2Brandom*%2C+EurekAlert&col=ev3rel&n=101&la=en) | Bosch MM, Barthelmes D, Merz TM, Knecht PB, Truffer F, Bloch KE, et al. New insights into changes in corneal thickness in healthy mountaineers during a very-high-altitude climb to Mount Muztagh Ata. Arch. Ophthalmol. 2010 févr;128(2):184-189. |
| 47 | [Medication appears well-tolerated, beneficial in Huntington's disease patients](http://ekasearch01.eurekalert.org/e3/cs.html?url=http%3A//www.eurekalert.org/pub_releases/2010-02/jaaj-maw020410.php&charset=iso-8859-1&qt=%2Brandom*%2C+EurekAlert&col=ev3rel&n=102&la=en) | Kieburtz K, McDermott MP, Voss TS, Corey-Bloom J, Deuel LM, Dorsey ER, et al. A randomized, placebo-controlled trial of latrepirdine in Huntington disease. Arch. Neurol. 2010 févr;67(2):154-160. |
| 48 | [Promising results shown for kidney cancer drug](http://ekasearch01.eurekalert.org/e3/cs.html?url=http%3A//www.eurekalert.org/pub_releases/2010-02/shro-prs020310.php&charset=iso-8859-1&qt=%2Brandom*%2C+EurekAlert&col=ev3rel&n=112&la=en) | Sternberg CN, Davis ID, Mardiak J, Szczylik C, Lee E, Wagstaff J, et al. Pazopanib in locally advanced or metastatic renal cell carcinoma: results of a randomized phase III trial. J. Clin. Oncol. 2010 févr 20;28(6):1061-1068 |
| 49 | [Dietary supplement speeds silver cyclists](http://ekasearch01.eurekalert.org/e3/cs.html?url=http%3A//www.eurekalert.org/pub_releases/2010-03/bc-dss032210.php&charset=iso-8859-1&qt=%2Brandom*%2C+%2Btype%3A"research"%2C+EurekAlert&col=ev3rel&n=2&la=en) | Chen S, Kim W, Henning SM, Carpenter CL, Li Z. Arginine and antioxidant supplement on performance in elderly male cyclists: a randomized controlled trial. J Int Soc Sports Nutr. 2010;7:13. |
| 50 | [Study shows phyical therapy exercise program can reduce risk of postnatal depression in new ...](http://ekasearch01.eurekalert.org/e3/cs.html?url=http%3A//www.eurekalert.org/pub_releases/2010-03/apta-ssp032210.php&charset=iso-8859-1&qt=%2Brandom*%2C+%2Btype%3A"research"%2C+EurekAlert&col=ev3rel&n=5&la=en) | Norman E, Sherburn M, Osborne RH, Galea MP. An exercise and education program improves well-being of new mothers: a randomized controlled trial. Phys Ther. 2010 mars;90(3):348-355 |
| 51 | [Fibromyalgia symptoms improved by lifestyle adjustments](http://ekasearch01.eurekalert.org/e3/cs.html?url=http%3A//www.eurekalert.org/pub_releases/2010-03/bc-fsi032610.php&charset=iso-8859-1&qt=%2Brandom*%2C+%2Btype%3A"research"%2C+EurekAlert&col=ev3rel&n=9&la=en) | Fontaine KR, Conn L, Clauw DJ. Effects of lifestyle physical activity on perceived symptoms and physical function in adults with fibromyalgia: results of a randomized trial. Arthritis Res. Ther. 2010;12(2):R55 |
| 52 | [Advance care planning improves end of life care and reduces stress for relatives](http://ekasearch01.eurekalert.org/e3/cs.html?url=http%3A//www.eurekalert.org/pub_releases/2010-03/bmj-acp032210.php&charset=iso-8859-1&qt=%2Brandom*%2C+EurekAlert&col=ev3rel&n=13&la=en) | Detering KM, Hancock AD, Reade MC, Silvester W. The impact of advance care planning on end of life care in elderly patients: randomised controlled trial. BMJ. 2010;340:c1345 |
| 53 | [Mipomersen offers new therapeutic strategy for inherited high cholesterol](http://ekasearch01.eurekalert.org/e3/cs.html?url=http%3A//www.eurekalert.org/pub_releases/2010-03/l-mon031110.php&charset=iso-8859-1&qt=%2Brandom*%2C+EurekAlert&col=ev3rel&n=17&la=en) | Raal FJ, Santos RD, Blom DJ, Marais AD, Charng M-J, Cromwell WC, et al. Mipomersen, an apolipoprotein B synthesis inhibitor, for lowering of LDL cholesterol concentrations in patients with homozygous familial hypercholesterolaemia: a randomised, double-blind, placebo-controlled trial. Lancet. 2010 mars 20;375(9719):998-1006. |
| 54 | [Magnetic stimulation offers potential nondrug treatment option for migraine patients](http://ekasearch01.eurekalert.org/e3/cs.html?url=http%3A//www.eurekalert.org/pub_releases/2010-03/l-mso030210.php&charset=iso-8859-1&qt=%2Brandom*%2C+EurekAlert&col=ev3rel&n=22&la=en) | Lipton RB, Dodick DW, Silberstein SD, Saper JR, Aurora SK, Pearlman SH, et al. Single-pulse transcranial magnetic stimulation for acute treatment of migraine with aura: a randomised, double-blind, parallel-group, sham-controlled trial. Lancet Neurol. 2010 avr;9(4):373-380. |
| 55 | [Octreotide acetate does not prevent treatment-induced diarrhea in anorectal cancer](http://ekasearch01.eurekalert.org/e3/cs.html?url=http%3A//www.eurekalert.org/pub_releases/2010-03/jotn-oad032210.php&charset=iso-8859-1&qt=%2Brandom*%2C+EurekAlert&col=ev3rel&n=25&la=en) | Zachariah B, Gwede CK, James J, Ajani J, Chin LJ, Donath D, et al. Octreotide acetate in prevention of chemoradiation-induced diarrhea in anorectal cancer: randomized RTOG trial 0315. J. Natl. Cancer Inst. 2010 avr 21;102(8):547-556. |
| 56 | [Integrated care can cut chronic back pain work disability by 4 months](http://ekasearch01.eurekalert.org/e3/cs.html?url=http%3A//www.eurekalert.org/pub_releases/2010-03/bmj-icc031510.php&charset=iso-8859-1&qt=%2Brandom*%2C+EurekAlert&col=ev3rel&n=32&la=en) | Lambeek LC, van Mechelen W, Knol DL, Loisel P, Anema JR. Randomised controlled trial of integrated care to reduce disability from chronic low back pain in working and private life. BMJ. 2010;340:c1035 |
| 57 | [Heat therapy shown effective in treating cutaneous leishmaniasis among US soldiers in Iraq](http://ekasearch01.eurekalert.org/e3/cs.html?url=http%3A//www.eurekalert.org/pub_releases/2010-03/plos-hts030210.php&charset=iso-8859-1&qt=%2Brandom*%2C+EurekAlert&col=ev3rel&n=35&la=en) | Aronson NE, Wortmann GW, Byrne WR, Howard RS, Bernstein WB, Marovich MA, et al. A randomized controlled trial of local heat therapy versus intravenous sodium stibogluconate for the treatment of cutaneous Leishmania major infection. PLoS Negl Trop Dis. 2010;4(3):e628. |
| 58 | [Acupuncture may relieve joint pain caused by some breast cancer treatments](http://ekasearch01.eurekalert.org/e3/cs.html?url=http%3A//www.eurekalert.org/pub_releases/2010-03/cumc-amr030410.php&charset=iso-8859-1&qt=%2Brandom*%2C+EurekAlert&col=ev3rel&n=37&la=en) | Crew KD, Capodice JL, Greenlee H, Brafman L, Fuentes D, Awad D, et al. Randomized, blinded, sham-controlled trial of acupuncture for the management of aromatase inhibitor-associated joint symptoms in women with early-stage breast cancer. J. Clin. Oncol. 2010 mars 1;28(7):1154-1160. |
| 59 | [Mount Sinai finds meta-cognitive therapy more effective for adult ADHD patients](http://search.eurekalert.org/e3/cs.html?url=http%3A//www.eurekalert.org/pub_releases/2010-03/tmsh-msf033010.php&charset=iso-8859-1&qt=Mount+Sinai+finds+meta-cognitive+therapy+more+effective+for+adult+ADHD+patients&col=ev3rel&n=1&la=en) | Solanto MV, Marks DJ, Wasserstein J, Mitchell K, Abikoff H, Alvir JM, Kofman MD. Efficacy of meta-cognitive therapy for adult ADHD.Am J Psychiatry. 2010 Aug;167(8):958-68. Epub 2010 Mar 15. |
| 60 | [Omega 3 curbs precancerous growths in those prone to bowel cancer](http://ekasearch01.eurekalert.org/e3/cs.html?url=http%3A//www.eurekalert.org/pub_releases/2010-03/bmj-o3c031710.php&charset=iso-8859-1&qt=%2Brandom*%2C+EurekAlert&col=ev3rel&n=74&la=en) | West NJ, Clark SK, Phillips RKS, Hutchinson JM, Leicester RJ, Belluzzi A, et al. Eicosapentaenoic acid reduces rectal polyp number and size in familial adenomatous polyposis. Gut. 2010 juill;59(7):918-925. |
| 61 | [Boston Medical Center partners with Engineered Care to reduce hospital readmissions](http://ekasearch01.eurekalert.org/e3/cs.html?url=http%3A//www.eurekalert.org/pub_releases/2010-03/bumc-bmc031610.php&charset=iso-8859-1&qt=%2Brandom*%2C+EurekAlert&col=ev3rel&n=76&la=en) | Jack BW, Chetty VK, Anthony D, Greenwald JL, Sanchez GM, Johnson AE, et al. A reengineered hospital discharge program to decrease rehospitalization: a randomized trial. Ann. Intern. Med. 2009 févr 3;150(3):178-187. |
| 62 | [Sirolimus-eluting stent better than zotarolimus-eluting stent in everyday clinical practice](http://ekasearch01.eurekalert.org/e3/cs.html?url=http%3A//www.eurekalert.org/pub_releases/2010-03/l-ssb031110.php&charset=iso-8859-1&qt=%2Brandom*%2C+EurekAlert&col=ev3rel&n=83&la=en) | Rasmussen K, Maeng M, Kaltoft A, Thayssen P, Kelbaek H, Tilsted HH, et al. Efficacy and safety of zotarolimus-eluting and sirolimus-eluting coronary stents in routine clinical care (SORT OUT III): a randomised controlled superiority trial. Lancet. 2010 mars 27;375(9720):1090-1099. |
| 63 | [Pioneering treatment reduces disability in premature babies with serious brain hemorrhage](http://ekasearch01.eurekalert.org/e3/cs.html?url=http%3A//www.eurekalert.org/pub_releases/2010-03/uob-ptr030510.php&charset=iso-8859-1&qt=%2Brandom*%2C+EurekAlert&col=ev3rel&n=104&la=en) | Whitelaw A, Jary S, Kmita G, Wroblewska J, Musialik-Swietlinska E, Mandera M, et al. Randomized trial of drainage, irrigation and fibrinolytic therapy for premature infants with posthemorrhagic ventricular dilatation: developmental outcome at 2 years. Pediatrics. 2010 avr;125(4):e852-858. |
| 64 | [Safety data favor norepinephrine over dopamine for shock](http://ekasearch01.eurekalert.org/e3/cs.html?url=http%3A//www.eurekalert.org/pub_releases/2010-03/eu-sdf030310.php&charset=iso-8859-1&qt=%2Brandom*%2C+EurekAlert&col=ev3rel&n=113&la=en) | De Backer D, Biston P, Devriendt J, Madl C, Chochrad D, Aldecoa C, et al. Comparison of dopamine and norepinephrine in the treatment of shock. N. Engl. J. Med. 2010 mars 4;362(9):779-789 |
| 65 | [Pneumococcal vaccine offers protection to HIV-infected African adults in clinical trial](http://ekasearch01.eurekalert.org/e3/cs.html?url=http%3A//www.eurekalert.org/pub_releases/2010-03/wt-pvo030110.php&charset=iso-8859-1&qt=%2Brandom*%2C+EurekAlert&col=ev3rel&n=116&la=en) | French N, Gordon SB, Mwalukomo T, White SA, Mwafulirwa G, Longwe H, et al. A trial of a 7-valent pneumococcal conjugate vaccine in HIV-infected adults. N. Engl. J. Med. 2010 mars 4;362(9):812-822. |
| 66 | [Study shows pine bark reduces blood pressure, counteracts kidney damage caused by ...](http://ekasearch01.eurekalert.org/e3/cs.html?url=http%3A//www.eurekalert.org/pub_releases/2010-03/mg-ssp030310.php&charset=iso-8859-1&qt=%2Brandom*%2C+EurekAlert&col=ev3rel&n=117&la=en) | Cesarone MR, Belcaro G, Stuard S, Schönlau F, Di Renzo A, Grossi MG, et al. Kidney flow and function in hypertension: protective effects of pycnogenol in hypertensive participants--a controlled study. J. Cardiovasc. Pharmacol. Ther. 2010 mars;15(1):41-46. |
| 67 | [Aspirin use does not significantly reduce events among those identified by certain ...](http://ekasearch01.eurekalert.org/e3/cs.html?url=http%3A//www.eurekalert.org/pub_releases/2010-03/jaaj-aud022510.php&charset=iso-8859-1&qt=%2Brandom*%2C+EurekAlert&col=ev3rel&n=120&la=en) | Fowkes FGR, Price JF, Stewart MCW, Butcher I, Leng GC, Pell ACH, et al. Aspirin for prevention of cardiovascular events in a general population screened for a low ankle brachial index: a randomized controlled trial. JAMA. 2010 mars 3;303(9):841-848 |
| 68 | [Office-based ultrasound-guided FNA superior in diagnosing head and neck lesions](http://ekasearch01.eurekalert.org/e3/cs.html?url=http%3A//www.eurekalert.org/pub_releases/2010-03/aaoo-ouf022310.php&charset=iso-8859-1&qt=%2Brandom*%2C+EurekAlert&col=ev3rel&n=131&la=en) | Robitschek J, Straub M, Wirtz E, Klem C, Sniezek J. Diagnostic efficacy of surgeon-performed ultrasound-guided fine needle aspiration: a randomized controlled trial. Otolaryngol Head Neck Surg. 2010 mars;142(3):306-309. |
| 69 | [Deep brain stimulation reduces epileptic seizures in patients with refractory partial and ...](http://ekasearch01.eurekalert.org/e3/cs.html?url=http%3A//www.eurekalert.org/pub_releases/2010-03/w-dbs031610.php&charset=iso-8859-1&qt=%2Brandom*%2C+EurekAlert&col=ev3rel&n=138&la=en) | Fisher R, Salanova V, Witt T, Worth R, Henry T, Gross R, et al. Electrical stimulation of the anterior nucleus of thalamus for treatment of refractory epilepsy. Epilepsia. 2010 mai;51(5):899-908. |
| 70 | [Research findings expected to ease treatment of low neutrophil counts in cancer patients](http://ekasearch01.eurekalert.org/e3/cs.html?url=http%3A//www.eurekalert.org/pub_releases/2010-03/sjcr-rfe031010.php&charset=iso-8859-1&qt=%2Brandom*%2C+EurekAlert&col=ev3rel&n=145&la=en) | Spunt SL, Irving H, Frost J, Sender L, Guo M, Yang B-B, et al. Phase II, randomized, open-label study of pegfilgrastim-supported VDC/IE chemotherapy in pediatric sarcoma patients. J. Clin. Oncol. 2010 mars 10;28(8):1329-1336. |
