# Supplementary material for: Misrepresentation of Randomized Controlled Trials in Press Releases and News Coverage: A Cohort Study
Source: PLoS Med. 2012 Sep 11;9(9):e1001308. doi: 10.1371/journal.pmed.1001308 (PMC3439420; doi:10.1371/journal.pmed.1001308)
Supplement: Text S5 — Multivariate analysis of factors associated with “spin” in press releases. (DOC) [file pmed.1001308.s005.doc]

**Text S5: Multivariate analysis of factors associated with “spin” in press releases**

For the multivariable analysis, we choose a bootstrap model selection variable method; the multivariable model on the full set of variables was evaluated 1000 times on the 1000 bootstrap samples.

Variables with a p-value < 0.25 in the bivariate analysis were included in the 1000 multivariable models (journal, spin in the abstract conclusion, sample size (large/small)). For each variable, the frequency of a p-value <0.05 is calculated and reported on the enclosed barplot.

Variables identified as independent factors associated with spin in the press release (p<0.05) in at least 60% of the bootstrap samples were kept in the final multivariable model. The only variable kept is spin in the abstract conclusion.


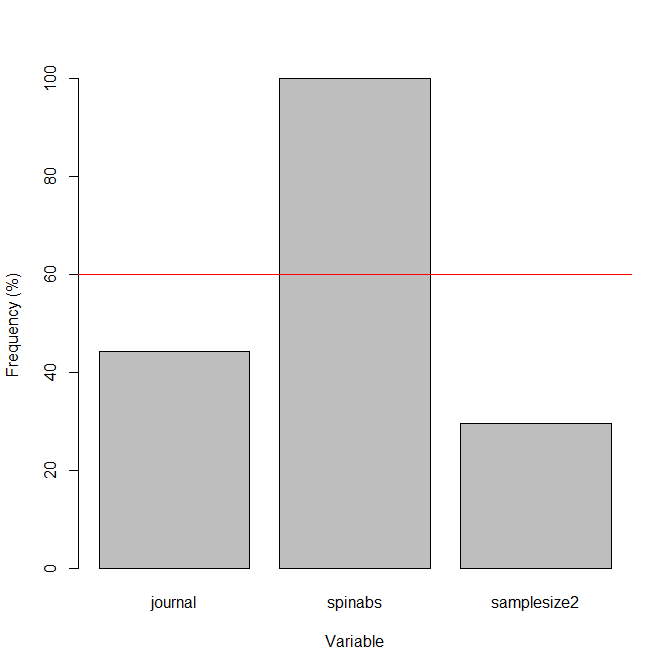


Journal

Sample size

Spin abstract
