# Supplementary material for: Misrepresentation of Randomized Controlled Trials in Press Releases and News Coverage: A Cohort Study
Source: PLoS Med. 2012 Sep 11;9(9):e1001308. doi: 10.1371/journal.pmed.1001308 (PMC3439420; doi:10.1371/journal.pmed.1001308)
Supplement: Text S6 — Bivariate analysis of factors associated with “spin” in news items (n = 41). (DOC) [file pmed.1001308.s006.doc]

**Text S6. Bivariate analysis of factors associated with “**spin” in news items (n = 41)

| Characteristics |  | Spin in the news | P value |
| --- | --- | --- | --- |
| Journal, n/total N (%) | General journal | 7/20 (35) | 0.043 |
|  | Specialized journal | 14/21 (67) |  |
| Sample size, n/total N (%) | Large | 6/19 (32) | 0.019 |
|  | Small | 15/22 (68) |  |
| Funding source, n/total N (%) | Profit or both | 13/21 (62) | 0.161 |
|  | Non-profit or not reported | 8/20 (40) |  |
| Experimental treatment, n/total N (%) | Drug | 12/24 (50) | 0.853 |
|  | Other | 9/17 (53) |  |
| Results of the primary outcome, n/total N (%) | All non-statistically significant | 6/15 (40) | 0.275 |
|  | Other | 15/26 (58) |  |
| Spin in abstract, n/total N (%) | ≥1 Spin | 17/17 (100) | <0.001 |
|  | No spin | 4/24 (5) |  |
| Spin in the press release, n/total N (%) | ≥1 Spin | 18/18 (100) | <0.001 |
|  | No spin | 3/23 (13) |  |
