# Supplementary material for: Misrepresentation of Randomized Controlled Trials in Press Releases and News Coverage: A Cohort Study
Source: PLoS Med. 2012 Sep 11;9(9):e1001308. doi: 10.1371/journal.pmed.1001308 (PMC3439420; doi:10.1371/journal.pmed.1001308)
Supplement: Text S7 — Bivariate analysis of factors associated with an overestimation of the benefit of the experimental treatment from the news as compared with the interpretation from the article abstract conclusions (n = 41). (DOC) [file pmed.1001308.s007.doc]

**Text S7. Bivariate analysis of factors associated with an overestimation of the benefit of the experimental treatment from the news as compared with the interpretation from the article abstract conclusions (n=41)**

| Characteristics |  | Overestimated from news items | P value |
| --- | --- | --- | --- |
| Journal, n/total N (%) | General journal | 2/20 (10) | 0.067 |
|  | Specialized journal | 8/21 (38) |  |
| Sample size, n/total N (%) | Large | 1/19 (5) | 0.011 |
|  | Small | 9/22 (41) |  |
| Funding source, n/total N (%) | Profit or both | 3/21 (14) | 0.159 |
|  | Non-profit or not reported | 7/20 (35) |  |
| Experimental treatment, n/total N (%) | Drug | 6/24 (25) | 1 |
|  | Other | 4/17 (24) |  |
| Results of the primary outcome, n/total N (%) | All non-statistically significant | 5/15 (33) | 0.453 |
|  | Other | 5/26 (19) |  |
| Spin in the news, n/total N (%) | ≥1 Spin | 9/21 (43) | 0.009 |
|  | No spin | 1/20 (5) |  |
